# Molecular Determinant Underlying Selective Coupling of Primary G‐Protein by Class A GPCRs

**DOI:** 10.1002/advs.202310120

**Published:** 2024-04-22

**Authors:** Qingya Shen, Xinyan Tang, Xin Wen, Shizhuo Cheng, Peng Xiao, Shao‐Kun Zang, Dan‐Dan Shen, Lei Jiang, Yanrong Zheng, Huibing Zhang, Haomang Xu, Chunyou Mao, Min Zhang, Weiwei Hu, Jin‐Peng Sun, Yan Zhang, Zhong Chen

**Affiliations:** ^1^ Department of Pharmacology and Department of Pathology of Sir Run Run Shaw Hospital & Liangzhu Laboratory Hangzhou 310058 China; ^2^ MOE Frontier Science Center for Brain Research and Brain‐Machine Integration Zhejiang University School of Medicine Hangzhou 310058 China; ^3^ Department of Pharmacology and Department of Pharmacy of the Second Affiliated Hospital NHC and CAMS Key Laboratory of Medical Neurobiology School of Basic Medical Sciences Zhejiang University School of Medicine Hangzhou 310058 China; ^4^ Advanced Medical Research Institute Meili Lake Translational Research Park Cheeloo College of Medicine Shandong University Jinan 250012 China; ^5^ Department of Biochemistry and Molecular Biology Shandong University School of Medicine Jinan 250012 China; ^6^ College of Computer Science and Technology Zhejiang University Hangzhou 310027 China; ^7^ Key Laboratory of Neuropharmacology and Translational Medicine of Zhejiang Province Zhejiang Chinese Medical University Hangzhou 310053 China; ^8^ Department of General Surgery Sir Run Run Shaw Hospital Zhejiang University School of Medicine Hangzhou Zhejiang 310016 China; ^9^ Zhejiang Research and Development Engineering Laboratory of Minimally Invasive Technology and Equipment Zhejiang University Hangzhou 310016 China; ^10^ Department of Physiology and Pathophysiology, School of Basic Medical Sciences Peking University Key Laboratory of Molecular Cardiovascular Science Ministry of Education Beijing 100191 China

**Keywords:** cryo‐EM structure, G protein selectivity, GPCR, H_2_R, H_3_R, machine learning, signaling complex

## Abstract

G‐protein‐coupled receptors (GPCRs) transmit downstream signals predominantly via G‐protein pathways. However, the conformational basis of selective coupling of primary G‐protein remains elusive. Histamine receptors H_2_R and H_3_R couple with G_s_‐ or G_i_‐proteins respectively. Here, three cryo‐EM structures of H_2_R‐G_s_ and H_3_R‐G_i_ complexes are presented at a global resolution of 2.6‐2.7 Å. These structures reveal the unique binding pose for endogenous histamine in H_3_R, wherein the amino group interacts with E206^5.46^ of H_3_R instead of the conserved D114^3.32^ of other aminergic receptors. Furthermore, comparative analysis of the H_2_R‐G_s_ and H_3_R‐G_i_ complexes reveals that the structural geometry of TM5/TM6 determines the primary G‐protein selectivity in histamine receptors. Machine learning (ML)‐based structuromic profiling and functional analysis of class A GPCR–G‐protein complexes illustrate that TM5 length, TM5 tilt, and TM6 outward movement are key determinants of the G_s_ and G_i/o_ selectivity among the whole Class A family. Collectively, the findings uncover the common structural geometry within class A GPCRs that determines the primary G_s_‐ and G_i/o_‐coupling selectivity.

## Graphical abstract

G‐protein‐coupled receptors (GPCRs) primarily signal through G‐protein pathways. Here, three cryo‐EM structures of H_2_R‐G_s_ and H_3_R‐G_i_ are reported at 2.6–2.7 Å resolution. Comparative analysis shows TM5/TM6 geometry determines primary G‐protein selectivity in histamine receptors. Machine learning profiling reveals TM5 length, TM5 tilt, and TM6 outward movement govern G_s_ and G_i/o_ selectivity in Class A GPCRs.

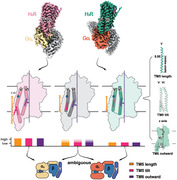

## 1 Introduction

Comprised of over 800 members, G‐protein‐coupled receptors (GPCRs) constitute the largest family of membrane proteins in the human genome.^[^ ^1^ ^]^ GPCR‐mediated signaling pathways are involved in virtually every physiological function and many pathologies, thus representing the targets of approximately one‐third of all medications, including antihistamines, antipsychotics, and opioid painkillers. In response to myriad extracellular stimuli, GPCRs exert adequate intracellular signaling predominantly through the engagement and activation of four major G‐protein families: G_s_, G_i/o_, G_q/11_, and G_12/13_.^[^ ^1^, ^2^ ^]^ Various G‐protein subtypes elicit distinct signaling pathways and trigger versatile physiological effects by regulating different key effectors, such as adenylate cyclase, phospholipase C, and mitogen‐activated protein kinases.^[^ ^2b^ ^]^ While GPCR activation by agonists typically triggers multiple G‐protein signaling pathways, many GPCRs exhibit pronounced bias and can preferentially activate certain downstream responses.^[^ ^3^ ^]^ In many circumstances, the beneficial effects of a drug come from one signaling pathway and the adverse effects from other pathways.^[^ ^4^ ^]^ Understanding the molecular mechanism underlying the selective coupling of an agonist‐stimulated GPCR to an appropriate G‐protein is therefore crucial for transmembrane signal transduction.

**Figure 1:**
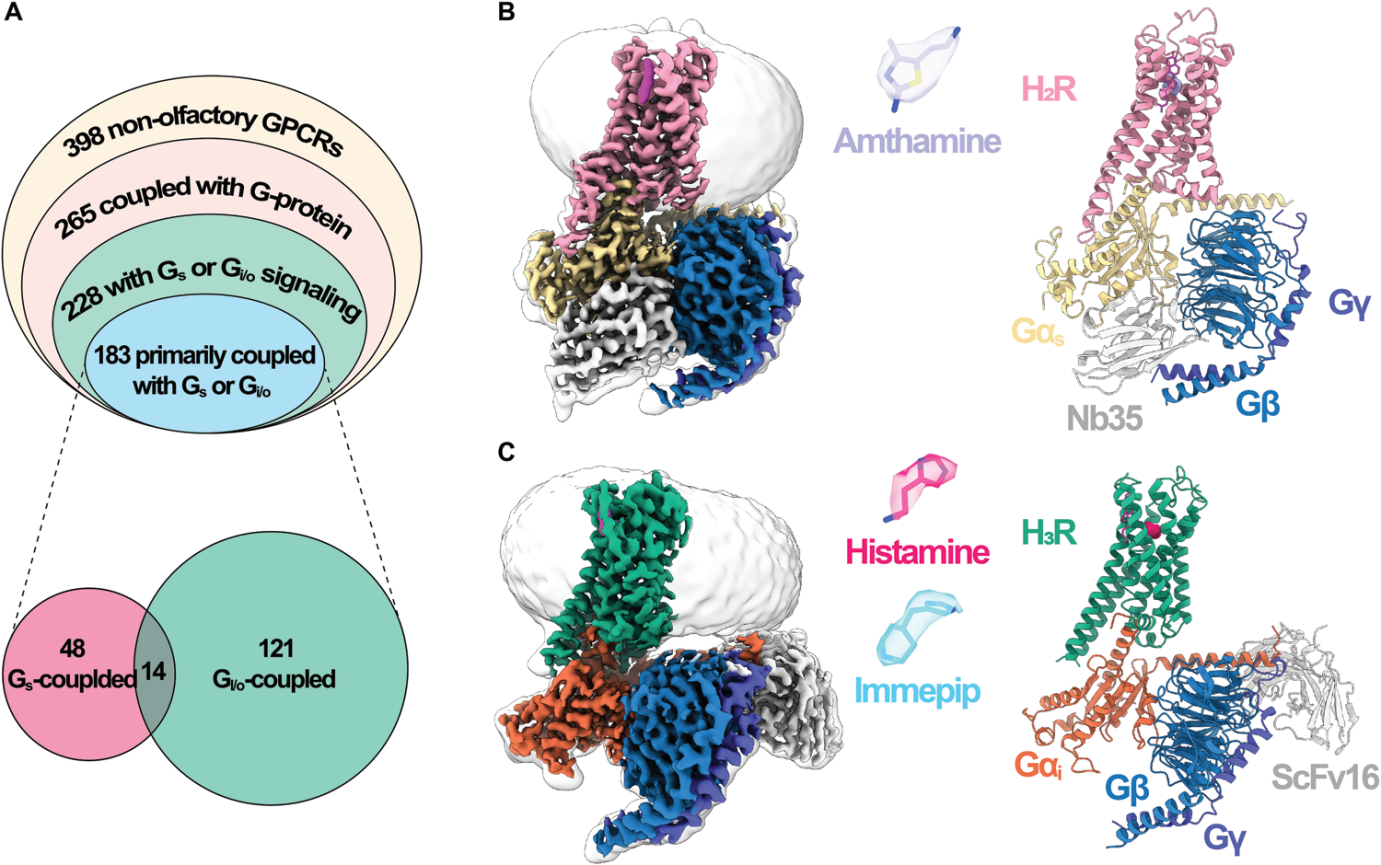
Cryo‐EM structures of H_2_R‐G_s_ and H_3_R‐G_i_ complexes. A) (Up) Proportion of G_s‐_ or G_i/o_‐coupled receptors in the non‐olfactory GPCRs. (Down) Number of receptors primarily coupling with G_s_‐ or G_i/o_‐proteins. B) The cryo‐EM density map (left) and atomic model (right) of amthamine‐bound H_2_R‐G_s_ complex. The amthamine is depicted as stick within a transparent EM density map. C) The cryo‐EM density map (left) and atomic model (right) of H_3_R‐G_i_ complex. The ligands histamine and immepip are depicted as sticks within a transparent EM density map.

An increasing number of studies suggested that many GPCRs exhibit a general promiscuity in their interactions with various G‐protein subfamilies.^[^ ^2^, ^5^ ^]^ The inherent dynamic nature of GPCRs enables them to sample multiple transition states between inactive and active states, contributing to the promiscuity of G‐protein selectivity.^[^ ^5^, ^6^ ^]^ Primary signaling stands out due to its remarkable coupling efficiency and rapid kinetics, emerging as the predominant signaling route for GPCRs among the spectrum of G‐protein signaling interactions. According to the GPCR database (GPCRdb), among 265 non‐olfactory GPCRs annotated on the G‐protein family level, 228 (86%) have the potential to modulate receptor‐specific cell activities by regulating adenylyl cyclase^[^ ^2^, ^5^, ^7^ ^]^ (**Figure** **1**A). Notably, among 183 GPCRs that predominantly couple with G_s_‐ or G_i/o_‐proteins, 169 GPCRs (92.3% of the total) exhibit a high degree of selectivity for either G_s_‐ (48 GPCRs) or G_i/o_‐ (121 GPCRs) proteins.^[^ ^2a^ ^]^ Therefore, G_s_‐ and G_i/o_‐proteins are the most prevalent G‐proteins among all the subtypes, leading to inverse cellular signaling responses through the stimulation or inhibition of adenylyl cyclase, respectively.

GPCR structures are indispensable tools, providing snapshots at local energy minimum that aid in uncovering the determinants for the primary signaling of G_s_‐ and G_i/o_‐protein selectivity.^[^ ^8^ ^]^ The resolution revolution in cryo‐electron microscopy (cryo‐EM) has led to an explosion of GPCR–G‐protein complex structures, particularly the G_s_‐ and G_i/o_‐coupled receptors. These structures have unveiled a common mode of GPCR–G‐protein recognition, in which the C‐terminal helix (α5) of Gα inserts into the cytosolic transmembrane (TM) cavity of GPCR.^[^ ^1^, ^9^ ^]^ The coupling determinants for GPCR in Gα proteins are widely accepted, with the selectivity barcode being the α5 helix, the αN‐β1 hinge, and the β2‐β3 loop of Gα.^[^ ^1^, ^9^ ^]^ However, the molecular basis of primary G‐protein‐coupling selectivity of GPCRs at the receptor side remains intricate. Most investigations suggest that GPCRs coupled with different G‐proteins generally adopt nearly identical conformations,^[^ ^10^ ^]^ with the exception of the 5HT_4_,^[^ ^11^ ^]^ resulting in the widespread doubts of the existence of complementary determinants at the GPCR level.^[^ ^12^ ^]^ Additionally, the absence of evolved consensus sequences for G‐protein recognition at GPCRs makes it difficult to draw a unified conclusion regarding primary G‐protein selectivity.^[^ ^1^, ^12^, ^13^ ^]^ Previous structural studies have underscored the importance of ICL2, ICL3, TM5, and TM6 of GPCRs in G_s_/G_i/o_‐protein selectivity.^[^ ^9^, ^14^ ^]^ However, the limitation is that these studies often focused on specific receptors or receptor subfamilies, and the observed characteristics are not universal across class A GPCR families, rendering the primary G‐protein selectivity mechanism still promiscuous and challenging.

Histamine, a biogenic amine, plays a pivotal role in various physiological and pathophysiological processes, such as allergic and inflammatory reactions, gastric acid secretion, wake‐sleep disorders, and neurotransmission in the central nervous system.^[^ ^15^ ^]^ The histamine receptor system comprises four members (H_1_R, H_2_R, H_3_R, and H_4_R), all of which belong to the Class A GPCR family.^[^ ^16^ ^]^ Drugs targeting H_1_R, H_2_R, and H_3_R have been approved to treat allergic disorders,^[^ ^17^ ^]^ gastroduodenal ulcers,^[^ ^18^ ^]^ and narcolepsy,^[^ ^19^ ^]^ respectively. The four subtypes of histamine receptors deliver downstream signaling by coupling with different heterotrimeric G‐proteins: H_1_R mainly couples with G_q_‐protein; H_2_R primarily couples with G_s_‐protein and could alternatively couple with G_q_‐protein; H_3_R and H_4_R are both capable of mediating downstream signaling via G_i/o_‐protein activation primarily. Given their diverse coupling profiles, histamine receptors serve as an excellent model system for investigating the structural basis of primary G‐protein‐coupling and the preferential profile of GPCRs.

To date, structures of the inactive states of H_1_R, H_2_R, and H_3_R, as well as the active states of H_1_R and H_4_R, are available.^[^ ^20^ ^]^ However, the absence of active‐state structures of H_2_R and H_3_R has impeded the understanding of the molecular basis of H_2_R and H_3_R signaling and the structural‐based drug design of histamine receptors. In this study, we report three cryo‐EM structures of H_2_R‐G_s_ and H_3_R‐G_i_ complexes at a global resolution of 2.6–2.7 Å. These structures reveal the structural basis for agonist recognition and G‐protein selectivity of H_2_R and H_3_R. A comparative analysis of the H_2_R‐G_s_ and H_3_R‐G_i_ complexes reveals that the conformation of TM5 and TM6 determines G‐protein‐selectivity in histamine receptors. Furthermore, machine learning (ML)‐based structural profiling and functional analysis of publicly available and home‐generated class A GPCR–G‐protein complexes indicate that TM5 length, TM5 tilt, and TM6 outward movement determine G_s_ and G_i/o_ selectivity. To further enhance our understanding, we develop a ML‐derived architecture classifier for G_s_‐ and G_i/o_‐coupled receptors using homology models from GPCRdb as a training dataset. Remarkably, the classifier achieves an accuracy up to 91%, as validated by experimentally determined GPCR‐G complex structures. Collectively, our results reveal the common structural geometries of class A GPCRs determine primary G_s_‐ and G_i/o_‐coupling selectivity.

## 2 Results

### 2.1 Cryo‐EM Structures of the Agonist‐Bound H_2_R‐G_s_ and H_3_R‐G_i_ Complexes

To investigate the structure of the H_2_R‐G_s_ complex, we co‐expressed full‐length H_2_R with the human heterotrimeric G_s_ protein in Sf9 insect cells. A flag tag was fused to the N‐terminus of H_2_R to aid purification. The H_2_R‐G_s_ complex was assembled in the presence of a highly selective agonist, amthamine, and further stabilized by a camelid antibody, Nb35.^[^ ^21^ ^]^ Utilizing single‐particle cryo‐EM analysis, the purified H_2_R‐G_s_ complex was solved at a nominal resolution of 2.7 Å (Figure 1B; Figures S1, S2 and Table S1, Supporting Information). The H_3_R‐G_i_ complex was obtained through a co‐expression strategy, similar to that of the H_2_R‐G_s_ complex. In addition to the N‐terminal flag tag, a C‐terminal tandem maltose‐binding protein (MBP) tag was also introduced to H_3_R. A NanoBiT tethering strategy was used to improve the stability of the H_3_R‐G_i_ complex.^[^ ^22^ ^]^ The endogenous agonist histamine and synthetic agonist immepip were used separately to generate the agonist‐bound H_3_R‐G_i_ complexes, and the complexes were further stabilized by a single‐chain antibody scFv16.^[^ ^23^ ^]^ The histamine‐bound H_3_R‐G_i_ complex and immepip‐bound H_3_R‐G_i_ complex were solved at a global resolution of 2.7 Å and 3.0 Å, respectively (Figure 1C; Figures S1,S2 and Table S1, Supporting Information).

All three GPCR–G‐protein complex structures are resolved at near‐atomic resolution, and the EM density maps were clear enough to build the model of the receptors, heterotrimeric G‐protein, antibody, and the bound ligand in the receptor orthosteric pockets (Figure S2 and Table S1, Supporting Information). The N‐ and C‐termini of both receptors, as well as the ICL3 of H_3_R and the α‐helical domains of Gα, exhibit poor resolution in the cryo‐EM density maps, consistent with previous cryo‐EM solved GPCR–G‐protein complexes due to the high flexibility of these regions. Notably, the density of cholesterol between TM1 and TM7 is observed in both H_2_R‐G_s_ and H_3_R‐G_i_ complexes, highlighting the critical role of cholesterol molecules in maintaining conformational stability and signaling activity of GPCR.

### 2.2 Ligand Recognition of Histamine Receptors

**Figure 2:**
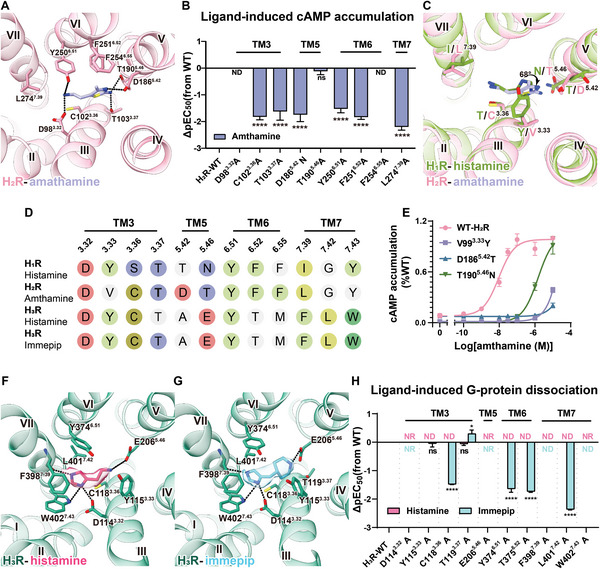
Ligand recognition of histamine receptors. A) Detailed interaction of amthamine with H_2_R. Dashed lines represent hydrogen bonds. B) Amthamine induced cAMP accumulation in HEK293 cells expressing H_2_R mutants of the residues in orthosteric pocket (*n* = 3, ordinary one‐way ANOVA, ****P < 0.0001, ND, not determinable, which refers to cannot be established over the tested concentration range, ns refers to no significance between the WT and mutant). C) Structural comparisons of ligand recognition between histamine‐H_1_R and amthamine‐H_2_R structures. D) Sequence alignment of the orthosteric pocket of histamine receptors. Residues that interact with ligands are highlighted with colored circles. E) Dose‐dependent curves for amthamine induced cAMP accumulation in HEK293 cells expressing the H_2_R mutants (V99^3.33^Y, D186^5.42^T, T190^5.46^N) that are not conserved in H_1_R (*n*  = 3). F‐G) Detailed interactions of histamine F) and immepip G) with H_3_R. H) Agonists induced G_i_ dissociation in HEK293 cells expressing H_3_R mutants of the residues in orthosteric pocket by NanoBiT assays (*n* = 3, ordinary one‐way ANOVA, *P = 0.0456, ****P < 0.0001, NR refers to no response to the ligand, ND, not determinable, which refers to cannot be established over the tested concentration range, ns refers to no significance between the WT and mutant).

In the H_2_R‐G_s_ complex, the agonist amthamine occupies the conventional orthosteric pocket of H_2_R. As depicted in Figure S3A (Supporting Information), this pocket is defined by TM3, 5, 6, and 7, and it is capped by the extracellular loop 2 (ECL2) of H_2_R. The binding of amthamine is stabilized through a combination of polar and hydrophobic interactions (**Figure** **2**A). Specifically, the aminoethyl group of amthamine is positioned between D98^3.32^ and Y250^6.51^ (we use the Ballesteros–Weinstein numbering system^[^ ^24^ ^]^), engendering a charge‐charge interaction with D98^3.32^— a conserved “salt‐bridge” also observed in other available monoamine receptor structures (Figure 2A).^[^ ^20^, ^25^ ^]^ Additionally, the two amines present in the thiazolamine moiety of amthamine establish polar interactions with T103^3.37^, D186^5.42^, and T190^5.46^ of H_2_R (Figure 2A). Consequently, the polar network effectively restrains both the aminoethyl group (head) and thiazolamine group (tail) of amthamine. Consistent with these observations, alanine mutagenesis of the majority of these residues markedly attenuated the cAMP accumulation response of H_2_R to amthamine (Figure 2B; Figure S4A and Table S2, Supporting Information). It's important to note that the T190^5.46^A mutant exhibits an anomalous behavior, possibly attributed to compensatory effects involving T103^3.37^. The thiazole ring of amthamine is sandwiched between V99^3.33^ on one side and F251^6.52^/F254^6.55^ on the other side, facilitating crucial hydrophobic interactions (Figure 2A). The methyl group position on the thiazole ring intensifies the hydrophobic interaction with F254^6.55^. Moreover, amthamine establishes van der Waals interactions with C102^3.36^ and L274^7.39^ (Figure 2A). Our mutational studies targeting these residues corroborate the substantial import of these hydrophobic interactions (Figure 2B; Figure S4A and Table S2, Supporting Information).

H_2_R displays the greatest similarity to H_1_R among the four histamine receptors, exhibiting an approximate 73% sequence homology in the 7TM region. As anticipated, the residues within the binding pocket of both H_1_R and H_2_R manifest a high degree of conservation. Among the 12 residues within the H_2_R binding pocket, seven are identical and three show homogeneous to their counterparts in H_1_R (Figure 2D; Figure S3F, Supporting Information). Notably, despite this conservation, discernible differences emerge in the ligand pocket shapes of H_1_R and H_2_R (Figure S3B,C, Supporting Information). Y108^3.33^ and N198^5.46^, possessing substantial steric bulk, serve to constrict the ligand pocket in H_1_R (Figure 2C). In contrast, the structurally equivalent residues V99^3.33^ and T190^5.46^ in H_2_R do not introduce such steric hindrance. As a consequence, the docking of amthamine into H_1_R would inevitably give rise to significant steric clashes, particularly involving the thiazole ring and the primary amine moiety of the ring with Y108^3.33^ and N198^5.46^ (Figure 2C). Furthermore, the substitution of D186^5.42^ in H_2_R with the corresponding T194^5.42^ residue in H_1_R diminished the polar interactions with amthamine (Figure 2C). Collectively, our structures provide a clear explanation for the high selectivity of amthamine as an H_2_R agonist. Furthermore, mutations of these three residues of H_2_R to the corresponding residues in H_1_R significantly decreased the potency and efficacy of amthamine, further supporting our conclusion (Figure 2E; Table S2, Supporting Information).

The H_3_R‐G_i_ complexes, bound with histamine and immepip, exhibit highly analogous overall structures, featuring a remarkably low root‐mean‐square deviation (RMSD) of 0.3 Å for the H_3_R Cα atoms. Both ligands reside within the orthosteric pocket, a characteristic shared with H_2_R and other aminergic receptors, as depicted in Figure 2. In the histamine‐bound H_3_R‐G_i_ complex, the amino group of histamine engages a pivotal hydrogen bond with E206^5.46^ (Figure 2F). Simultaneously, the N^π^ atom within the imidazole ring establishes van der Waals forces with C118^3.36^, while also forming hydrogen bonds with D114^3.32^ and W402^7.43^ (Figure 2F). Furthermore, the N^τ^ atom establishes a hydrogen bond with the backbone of F398^7.39^ (Figure 2F). Through diligent alanine mutagenesis investigations, we affirmatively ascertained the indispensability of these interactions for histamine binding and the ensuing receptor activation. Notably, the alanine mutations targeting E206^5.46^, D114^3.32^, and W402^7.43^ unequivocally abolish the capacity for G_i_ protein activation (Figure 2H; Figure S4B and Table S2, Supporting Information). Additionally, the EC50 value of the C118^3.36^A mutant experiences an approximately 150‐fold reduction in comparison to wild‐type H_3_R. Furthermore, the imidazole ring of histamine securely embeds itself within the hydrophobic core constituted by Y374^6.51^, L401^7.42^, W402^7.43^, and F398^7.39^ (Figure 2F). This interaction is particularly evident in the formation of a face‐to‐face π‐π stacking association with F398^7.39^. The veracity of these observations is endorsed by alanine substitutions targeting the residues implicated in this critical hydrophobic core, further underscoring their pivotal role in facilitating histamine recognition (Figure 2H; Figure S4B and Table S2, Supporting Information).

Within the H_3_R orthosteric pocket, the imidazole rings of histamine and immepip overlap and share many interactions with H_3_R in a nearly identical position (Figure 2G). Immepip, a synthetic agonist derived from histamine, replaces the aminoethyl moiety with a piperidine ring. A notable departure emerges in the positioning of the amine within the piperidine ring, situated a mere 1.3 Å closer to E206^5.46^ than the primary amine in histamine, thus intensifying its polar interactions with E206^5.46^. The bulky piperidine ring also establishes hydrophobic and van der Waals interactions with Y115^3.33^ and T119^3.37^, conferring enhanced compatibility of immepip with the dumbbell‐shaped pocket characteristic of H_3_R (Figure S3D,E, Supporting Information). Consequently, the binding interface of immepip with H_3_R spans ≈290 Å^2^, an appreciable 20 Å^2^ more expansive than that of histamine. Notably, despite modifications introduced to Y115^3.33^ and T119^3.37^, the resulting effect on the pEC_50_ values to immepip is negligible, potentially attributable to the compensatory amplification of polar interactions between the piperidine moiety and E206^5.46^ (Figure 2H; Figure S4C and Table S2, Supporting Information).

### 2.3 Unique Histamine Binding Mode of H_3_R

**Figure 3:**
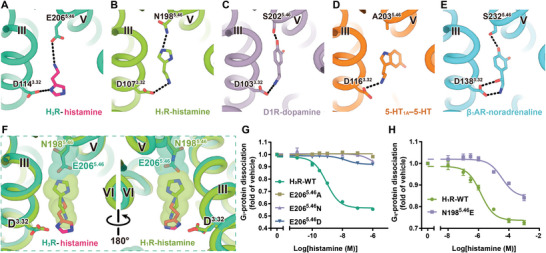
Endogenous ligand binding poses of aminergic receptors. A–E) Binding poses of H_3_R (A), H_1_R (B), D1R (C), 5‐HT_1A_ (D), and β_1_AR (E) with its endogenous ligands. F) Superimposition of H_3_R‐hisatime and H_1_R‐histamine structures. G,H) Effects of histamine‐induced G‐protein dissociation in HEK293 cells expressing histamine receptor mutants at 5.46 position. Dose‐response curves for H_3_R E206^5.46^ mutants (G) and H_1_R N196^5.46^E mutant (H) were measured using the NanoBiT assay (*n*  = 3).

Recent reports have detailed the structures of aminergic receptors bound to their endogenous ligands, including H_1_R with histamine,^[^ ^20a^ ^]^ D1R with dopamine,^[^ ^25c^ ^]^ 5‐HT_1A_ with 5‐HT,^[^ ^25a^ ^]^ and β_1_AR with noradrenaline.^[^ ^26^ ^]^ Comparison of these structures reveals that the biogenic monoamine ligands adopt a nearly identical binding pose, situated at the bottom of the receptor's orthosteric binding pockets. Notably, the amino group of these monoamine ligands forms a salt‐bridge with the residue D^3.32^, which is highly conserved among aminergic receptors. Intriguingly, our high‐resolution cryo‐EM Map unveils a distinctive binding pose of histamine in H_3_R, in which the amino group of histamine forms a salt‐bridge with E206^5.46^ rather than interacting with D114^3.32^ in other aminergic receptors (**Figure** **3**A–F; Figure S5, Supporting Information). To further validate this surprising observation, we constructed an alternative model with histamine adopting the classical pose observed in other aminergic receptors. This alternative model, along with the non‐classical model revealed by cryo‐EM map, were subjected to molecular dynamics (MD) simulation. As anticipated, histamine in our non‐classical pose for H_3_R remained stable throughout a 200 ns simulation (Figure S5B, Supporting Information). In contrast, histamine in the classic pose of aminergic receptors, as seen in H_1_R, exhibited high instability after a 200 ns simulation, with two considerably distinct conformations in the trajectory (Figure S5C, Supporting Information). These findings conclusively establish that histamine adopts a unique pose in H_3_R compared to other aminergic receptors.

Sequence alignment of all aminergic receptors reveals that the 5.46 position is mainly occupied by small side‐chain residues that are either uncharged polar or hydrophobic, such as threonine, serine, and alanine (Table S3, Supporting Information). This allows sufficient space to accommodate the bulky conjugated group of monoamine ligands. Consequently, the relatively compact imidazole group of histamine enables the inclusion of residues with bulkier side chains in the orthosteric binding pocket, as exemplified by the presence of asparagine in H_1_R. However, compared to A^5.46^ in 5‐HT_1A_ and S^5.46^ in D1R, the elongated glutamine residue in H_3_R causes severe steric hindrance with the imidazole group in the classic pose. Consequently, histamine rotates 180‐degree along the bilayer plane, resulting in the amino group of histamine interacting with E206^5.46^ instead of D114^3.32^ (Figure 3A–F; Figure S5, Supporting Information). A comparison of the orthosteric ligand binding pockets of H_1_R and H_3_R reveals that five of the eight residues constituting the H_3_R ligand pocket are not conserved with H_1_R (Figure 2D; Figure S3F, Supporting Information). This indicates that the shape and size of histamine binding pockets are significantly distinct between the two histamine receptors. The unique histamine binding of H_3_R is further confirmed by the differential effects of mutations of H_1_R and H_3_R at the 5.46 position (Figure 3G,H; Table S4, Supporting Information). This position contributes to the different binding capacities to the endogenous ligand histamine, enabling the bulk discovery of selective agonists and antagonists targeting H_3_R.

### 2.4 Structural Differences Reveal the Basis of G‐Protein Selectivity in Histamine Receptors

Histamine receptors H_1_R, H_2_R, and H_3_R primarily couple with G_q_‐, G_s_‐, or G_i_‐proteins, respectively. Our structures of H_2_R‐G_s_ and H_3_R‐G_i_ complexes, along with the recently determined H_1_R‐G_q_ structures,^[^ ^20a^ ^]^ enable structural comparisons among histamine receptor–G‐protein signaling complexes. Such comparative analysis holds the potential to unveil the intricate molecular determinants underlying G‐protein‐coupling preferences within this receptor subfamily, offering insights that could extend to other GPCRs. Upon superimposing the three structures focused on the receptor, we observe a congruence in the alignment of TM1, TM2, TM3, TM7, and ICL1 on the cytoplasmic side. Conversely, disparities emerge in the configuration of TM4‐6 and ICL2‐3 (Figure S6A,B, Supporting Information). This observation highlights that while the α5 of Gα inserts into the intracellular cavity of the receptors, contributing to the classical α5‐insertion interface within the complex structures, the specific manner in which the G‐protein binds diverges significantly across the three structures.

In both H_1_R‐G_q_ and H_3_R‐G_i_ structures, TM6 exhibits comparable outward displacements at their cytoplasmic ends. However, in the H_2_R‐G_s_ complex, a significant TM6 movement of 4.7 Å further outward than in H_3_R, as measured at Cα atoms of residue 6.30 (Figure S6C, Supporting Information). Notably, this represents the most significant TM6 outward displacement among the histamine receptor subfamilies. Consequently, the C‐terminus of α5 in Gα_s_ shifts by 4.0 Å toward TM6 in comparison to Gα_i_ within the H_3_R‐G_i_ complex, as measured at the conserved Cα atoms of residue H5.25 (common Gα numbering system)^[^ ^27^ ^]^ in Gα (Figure S6C, Supporting Information). Additionally, beyond the dynamic TM6, we observed significant variations in the conformation of TM4, as well as their corresponding ICL2 regions, on the cytoplasmic side. Notably, the intracellular tip of TM4 in H_3_R exhibits a noteworthy 4.9 Å shift toward TM2 in comparison to H_2_R, as determined by measuring the Cα atoms of residue 4.40 (Figure S6B, Supporting Information). This specific movement induces the ICL2 of H_3_R to rotate approximately 64‐degree compared to H_2_R (Figure S6B, Supporting Information). Besides, our H_2_R‐G_s_ and H_3_R‐G_i_ structures also reveal that the ICL2s form polar and hydrophobic interactions with the αN helix, the β2‐β3 loop, and the α5 helix of Gα, which are critical for G‐protein coupling.

**Figure 4:**
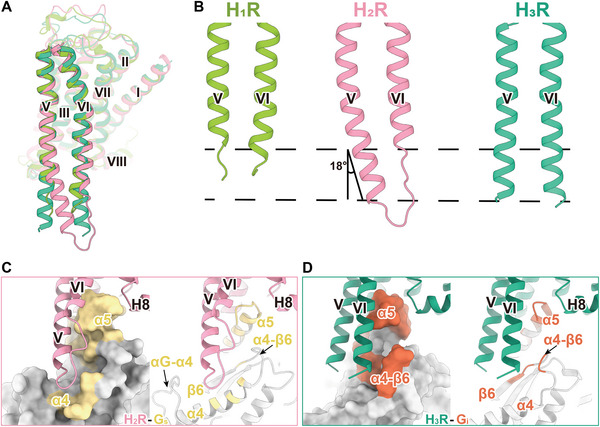
Structural comparison of histamine receptor–G‐protein complexes. A) Structural superposition of the active models of H_1_R, H_2_R, and H_3_R. B) Comparison of TM5 and TM6 in H_1_R‐G_q_, H_2_R‐G_s_, and H_3_R‐G_i_ complexes. C,D) Surface (left) and cartoon (right) representation of binding area of H_2_R (C) and H_3_R (D) with their respective coupled Gα subunit. Residues within 4Å of H_2_R or H_3_R in Gα_s_ or Gα_i_ are highlighted in yellow or orange, respectively.

The most notable distinctions among the three GPCR–G‐protein complexes are prominently evident within the TM5/TM6 regions (**Figure** **4**A,B). In the H_2_R‐G_s_ complex, the length TM5 of H_2_R extends ≈2.5 helical turns (from position 5.67 to 5.75, encompassing nine residues) compared with H_1_R (Figure 4B; Figure S2, Supporting Information). Furthermore, the extended TM5 in H_2_R tilts toward TM6 from the Y202^5.58^ residue for an 18‐degree angle (Figure 4B). Subsequently, the cytosolic tip of TM5 inserts into the cavity of Gα_s_, which is formed by the αG‐α4 loop, the α4 helix, and the α4‐β6 turn, resulting in additional interactions with the α4 of Gα_s_ (Figure 4C). In the H_3_R‐G_i_ structure, both TM5 and TM6 of H_3_R exhibit unexpected elongations comprising nine and eight residues (2.5 and 2.2 helical turns) compared to H_1_R, respectively (Figure 4B). This elongation of TM5/TM6 regions in H_3_R establishes close contacts with the α4‐β6 turn of Gα_i_, thereby strengthening the H_3_R‐G_i_ coupling effectiveness (Figure 4D). Consequently, the interfaces of H_2_R‐G_s_ (1610 Å^2^) and H_3_R‐G_i_ (1381 Å^2^) are both significantly larger than that of H_1_R‐G_q_ (1144 Å^2^). In conclusion, the elongations observed in TM5/TM6 regions within H_2_R and H_3_R constitute additional yet distinctive interfaces between the receptor and G‐proteins, hereafter referred to as non‐canonical receptor‐Gα interface. We anticipated that the differential non‐canonical receptor‐Gα interactions resulting from the extended TM5/TM6 could be the key determinants for the primary G_s_ or G_i/o_ selectivity. To validate our hypothesis, we analyzed the structural geometries of TM5/TM6 among available GPCR–G‐protein structures. This exploration aimed to unravel potential the correlations between the G_s_‐ or G_i/o_‐coupling selectivity within class A receptors.

### 2.5 TM5 and TM6 are Responsible for G_s_ and G_i/o_ Selectivity

**Figure 5:**
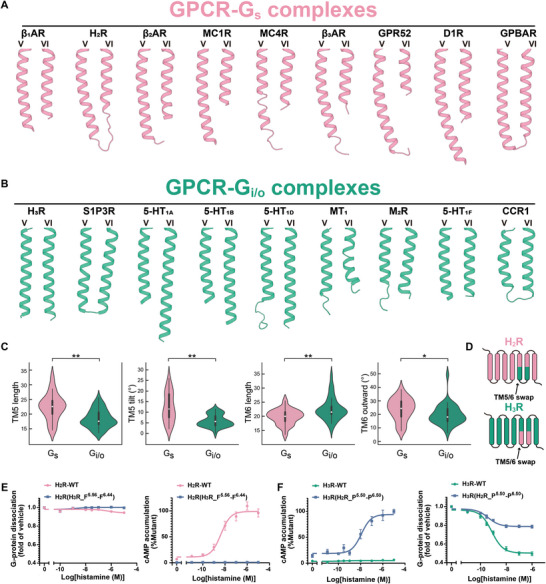
TM5 and TM6 are responsible for G_s_ and G_i_ selectivity. A) Representative of the TM5/TM6 structural geometry of G_s_‐coupled receptors. B) Representation of the TM5/TM6 structural geometry of G_i_‐coupled receptors. C) Violin plots depicting four distinct features of TM5/TM6 in G_s_‐ and G_i/o_‐coupled receptors. The white dot denotes the median. The interquartile range is shown by the broad black bar in the middle. Except for points considered to be “outliers” using an interquartile range‐based technique, the thin line reflects the remainder of the distribution. *n* = 54, ^ns^ *P* > 0.05, *P < 0.05, **P < 0.01 by Mann‐Whitney U test. D) Schematic representation of the “TM5/TM6 swap” experiments. E–F) Dose‐dependent curves for histamine induced G‐protein dissociation and cAMP accumulation in HEK293 cells expressing chimera receptors (*n*  = 3). E) Replacement of I^5.56^‐F^6.44^ of H_2_R with F^5.56^‐F^6.44^ of H_3_R did not confer the ability to dissociate G_i_‐protein (left), but resulted in loss of the ability to activate the G_s_ signal pathway (right). F) Replacement of P^5.50^‐P^6.50^ of H_3_R with P^5.50^‐P^6.50^ of H_2_R resulted in gain of the ability to activate the G_s_ signal pathway (left), but loss of the ability to dissociate G_i_‐protein (right).

Plentiful GPCR–G‐protein structures have been determined with increasing speed since we resolved the first two cryo‐EM structures of GPCR–G‐protein complexes in 2017.^[^ ^9a,b^ ^]^ Currently, ≈170 structures of class A GPCRs coupled to their corresponding G‐protein are available (as of July 2022). Among them, 13 G_s_‐ and 30 G_i/o_‐coupled signaling complex structures (Table S5, Supporting Information) of unique class A receptors, whose structures have been resolved with their corresponding primary G‐protein, are chosen for further analysis. We found that the extended TM5/TM6 structural geometries observed in H_2_R‐G_s_ and H_3_R‐G_i_ complexes are widespread in above structures. More than half of receptors in G_s_‐coupled state, the length of TM5 is remarkably longer than TM6 (the median length of TM5 and TM6 is 25 and 21, respectively (Table S5, Supporting Information). Helix lengths of the intracellular region are measured from the residue x.50 to the cytosolic tip of TM5 or TM6), including three β‐adrenergic receptors, D1R, MC1R, MC4R, GPBAR, GPR52, and H_2_R (**Figure** **5**A).^[^ ^14^, ^28^ ^]^ In addition, similar to H_2_R, the cytosolic halve of TM5 in G_s_‐coupled receptors exhibits a curved conformation toward TM6, with a range of tilting angles spanning from 8‐ to 26‐degree (Figure 5A; Table S5, Supporting Information). Nevertheless, the situation for G_i/o_‐coupled structures is more complex. For instance, certain receptors, including H_3_R, µOR, S1PR3, 5‐HT_1A_, 5‐HT_1B_, and 5‐HT_1D_, possess a TM6 length exceeding 26 residues.^[^ ^14^, ^25^, ^29^ ^]^ Notably, for 5‐HT_1A_, 5‐HT_1B_, and 5‐HT_1D_, the length of TM6 is 35, 31, and 31 residues, respectively. A significant proportion of GPCR‐G_i/o_ structures, including MT_1_, M_2_R, 5‐HT_1F_, chemokine receptors, etc., exhibit short helices of both TM5 and TM6, with the length of TM6 similar or only slightly longer than TM5 (Figure 5B).^[^ ^30^ ^]^ It is noteworthy that in the majority of G_i/o_‐coupled receptor structures, the TM5 is straight, as in H_3_R, indicating that the cytosolic ends of TM5 are not curved toward TM6, as in H_2_R in G_s_‐coupled structures (Figure 5A,B). Therefore, the length of TM5 and TM6, together with the tilt of the TM5 cytosolic end, exhibits significant differences between G_s_‐ and G_i/o_‐coupled receptors, likely responsible for G_s_ and G_i/o_ selectivity.

**Figure 6:**
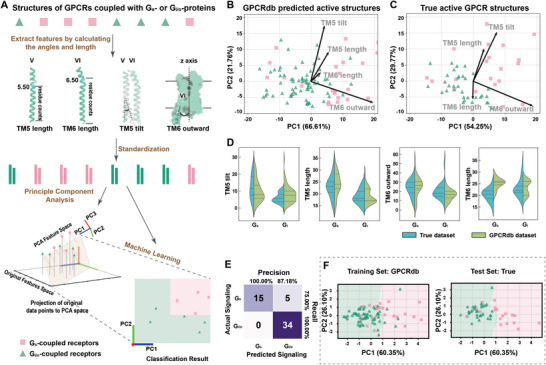
Machine learning‐based classification of GPCRs into G_s_ and G_i_ signaling pathway. A) Workflow for GPCR classification utilizing machine learning and feature pre‐processing. The length and tilt of TM5, as well as the length and outward movement of TM6 are extracted from active GPCR structures obtained from GPCRdb homology models and True structures. The resulting data of these four geometries are standardized, and then subjected to PCA. Subsequently, a Random Forest classifier is employed to classify GPCRs based on the PCA results. B,C) PCA biplots were generated for the four geometries of GPCRdb homology models (B) and True GPCR structures (C) individually. The contributions of each variable to the principal components are depicted as vectors on the plot, where the vertical component of a vector on a given PC illustrates the respective contribution of that variable to the PC. The angle between two vectors reflects the correlation between the corresponding features, while the length of a vector represents the significance of the corresponding feature. D) Comparison of violin plots depicting four geometries of TM5/TM6 in GPCRdb structures and True GPCR structures. Here the median and interquartile range are depicted as dashes. E) Confusion matrix for the True GPCR structures given by the best classifier. F) Decision boundary visualization via PCA given by the best classifier and scatter plot of GPCRdb homology models (left) and True GPCR structures (right) based on GPCRdb homology models.

Previous research has established that the outward movement of TM6 is the hallmark of receptor activation, thus plays a crucial role in receptor G_s_/G_i/o_‐coupling selectivity. Specifically, G_s_‐coupled structures exhibit larger outward movements of TM6 compared to G_i/o_‐coupled receptors.^[^ ^9c^ ^]^ To evaluate the significance of four structural geometries of TM5 and TM6 in G_s_ and G_i/o_ selectivity (the lengths of TM5 and TM6, the tilt of the TM5 cytosolic end, and the outward movement of TM6), we analyzed currently available G_s_‐ and G_i/o_‐coupled structures of class A receptors, along with our unpublished structures (including a total of 20 G_s_‐ and 34 G_i/o_‐coupled receptors) (Table S5, Supporting Information). The lengths of TM5 and TM6 were measured from the cytosolic tip to 5.50 and 6.50, respectively, and the lengths and angles of TM5/TM6 of receptors were extracted as illustrated in **Figure** **6**A (see Experimental Section). Consistent with our speculation, significant differences were observed in the geometries of TM5 and TM6 between G_s_‐ and G_i/o_‐coupled receptors. The average length of TM5 in G_s_‐coupled receptors is four residues longer than that of G_i/o_‐coupled receptors (23.1 ± 4.6 and 18.9 ± 3.1, respectively) (Figure 5C; Table S5, Supporting Information). The tilt of TM5 in G_s_‐coupled structures was distributed into two major clusters (12.3 ± 7.1°), while the majority of the G_i/o_‐coupled receptors exhibit smaller tilts (5.90 ± 2.9°) (Figure 5C; Table S5, Supporting Information). The average outward movement of TM6 in G_s_‐coupled receptors was slightly larger than that of G_i/o_‐coupled structures (23.8 ± 8.4° and 19.7 ± 7.6°, respectively), consistent with previous findings (Figure 5C; Table S5, Supporting Information). However, the TM6 lengths of G_s_‐coupled receptors and G_i/o_‐coupled receptors were indistinguishable, with comparable length distributions and medians (Figure 5C; Table S5, Supporting Information). In conclusion, the structural geometries of TM5/TM6, particularly the length of TM5, the tilt of cytosolic TM5, and the outward movement of TM6, may be responsible for primary G_s_ and G_i/o_ selectivity.

To validate the hypothesis that the preference selection of G‐proteins for class A receptors is determined by TM5/TM6, we conducted “TM5/TM6 swap” experiments to reverse the G_s_ and G_i/o_ selectivity of receptors (Figure 5D). In the first set of experiments, we replaced the intracellular half of TM5, TM6, and ICL3 (P^5.50^ to P^6.50^) of G_i/o_‐coupled receptors with that of G_s_‐coupled receptors. As expected, these chimeric receptors induced cAMP accumulation, including H_3_R(H_2_R_P^5.50^‐P^6.50^), H_3_R(D1R_P^5.50^‐P^6.50^), H_3_R(MC4R_P^5.50^‐P^6.50^), and H_3_R(β2AR_P^5.50^‐P^6.50^), and D2R(H_2_R_P^5.50^‐P^6.50^) (Figure 5E; Figure S7A and Table S6, Supporting Information). In the second set of experiments, we attempted to switch G_s_‐coupled receptors to G_i/o_‐coupled receptors. Although these engineered receptors could not recruit G_i_ proteins, their ability to stimulate cAMP accumulation was almost lost (Figure 5F; Figure S7B and Table S6, Supporting Information). In conclusion, these “TM5/TM6 swap” experiments could alter the G_s_‐ or G_i/o_‐coupling selectivity of receptors, supporting the notion that the conformational architecture of TM5/TM6 determines the G_s_ and G_i/o_ selectivity of a GPCR.

### 2.6 Classification of G_s_‐ and G_i/o_‐Coupled Receptors by Machine Learning

Can the structural determinants of G_s_ and G_i/o_ selectivity identified in this study be generalized to all Class A GPCRs? To address this question, a machine learning approach was utilized to categorize primary G_s_‐ and G_i/o_‐coupled receptors, based on the structural geometries of TM5/TM6 of receptors (Figure 6A). To augment the generalization of the predictive model, we incorporated predicted structures in the structural datasets (see Experimental Section). The training set comprised 98 (24 G_s_‐ and 74 G_i/o_‐coupled receptors) GPCRdb‐predicted homology models (Table S7, Supporting Information),^[^ ^31^ ^]^ while the test set consisted of 54 (20 G_s_‐ and 34 G_i/o_‐coupled receptors) true (experimental) structures (Table S5, Supporting Information). In light of potential correlations among the four structural geometries of TM5/TM6, we opted to apply Principal Component Analysis (PCA)^[^ ^32^ ^]^ to effectively decorrelate and compress these characterizes, thereby facilitating a reduction of the TM5/TM6 geometry space from a 4D to a 2D construct (Figure 6B,C). The first two Principle Component (PCs) are reserved for further analysis (PC1 and PC2), contributing more than 86% of the variance (Figure 6B,C). The individual biplots of the training and true datasets revealed that the TM5 length, TM5 tilt, and TM6 outward movement are the primary contributors to the principal components (The contributions of each variable to the principal components are depicted as vectors on the plot, where the vertical component of a vector on a given PC illustrates the respective contribution of that variable to the PC) (Figure 6B,C). Specifically, the TM6 outward movement significantly influences PC1, whereas the length and tilt of TM5 play a critical role in PC2 (Figure 6B,C). Moreover, these three vectors exhibit similarities in the principal component plots between the training and true datasets, particularly in terms of TM5 tilt and TM6 outward movement (Figure 6B,C). The significance of TM5 length, TM5 tilt, and TM6 outward movement in determining the selectivity of G_s_‐ and G_i/o_‐protein coupling in class A receptors is suggested by these findings.

Remarkably, the biplots indicate that between the two datasets, the TM6 length has the largest discrepancy: its eigenvector directions are almost opposite in the two biplots, whereas the remaining vectors have similar directions (Figure 6B,C). In the training dataset, the impact of the TM6 length vector on each principal component appears to be minimal and positively correlated to both PCs (Figure 6B,C). In contrast, it exerts a negligible effect on PC1 and displays a negative correlation with PC2 in the true dataset (Figure 6B,C). Subsequent violin plots reveal a symmetry of the distributions of TM5 tilt and TM6 outward movement in the two datasets (Figure 6D). Nevertheless, there is a notable disparity in the distribution pattern of TM6 length between the two datasets (Figure 6D). Further correlation analysis of four geometries of TM5/TM6 elucidates similar inconsistency. The TM6 length from the training dataset reveals a mildly positive correlation of 0.20 for the TM5 tilt (Figure S8, Supporting Information). Conversely, the TM6 length from the true dataset unveils a negative correlation for TM5 tilt, with a magnitude of −0.29 (Figure S8, Supporting Information). In conclusion, these results imply that the TM6 length poses a challenge for model training, concurrently indicating that TM6 length marginally contributes to G‐protein selectivity.

Aimed at deriving a more precise conclusion about the underlying mechanism, we trained a classifier for primarily G_s_‐ and G_i/o_‐coupled receptors through a machine‐learning approach directly on the three standardized geometries of TM5/TM6 (TM6 length excluded). We employed a variant of the Random Forest Classifier^[^ ^33^ ^]^ with constraints to the number of estimators and their maximum depth to decrease the risk of overfitting. Validation of the model using the true dataset achieved an accuracy of 90.74%, F1‐score of 0.8943, Area Under Curve (AUC) of 0.8162, and Matthews Correlation Coefficient (MCC) of 0.8086 (Figure 6E; Tables S5,S8, Supporting Information). This further illustrates the effectiveness of these three geometries for selectivity. Interestingly, certain falsely predicted receptors (PE2R2, PE2R4, V2R) in our classifier exhibited comparable G_s_ and G_i/o_‐protein selectivity, and LSHR had limited proof to support its primary coupling according to the GPCRdb/GproteinDb.^[^ ^31^ ^]^ In brief, the predictive model has fulfilled good performance.

**Figure 7:**
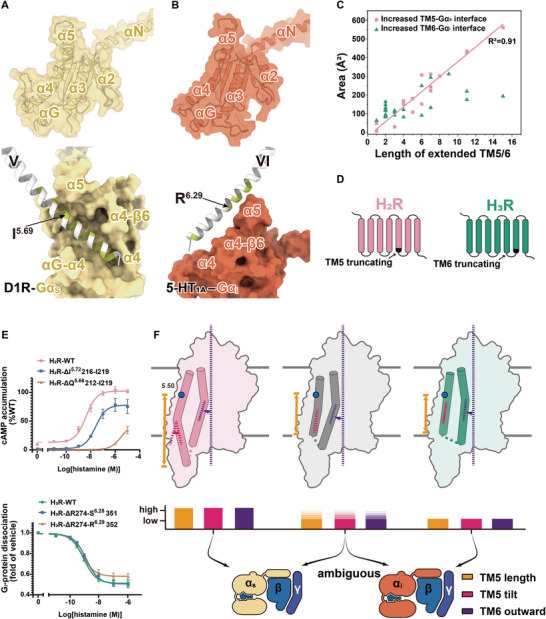
Contributions to G‐protein signaling of extended TM5/TM6. A) (Up) Surface and cartoon representations of Gα_s_ are shown, with the surface of Gα_s_ depicted as transparent. (Down)The binding surface of TM5 with Gα_s_ of D1R is shown, with TM5 beyond I^5.69^ noted as extended helix. Residues that interact with Gα are highlighted in green. B) (Up)Surface and cartoon representations of Gα_i_ are shown, with the surface of Gα_i_ depicted as transparent. (Down) The binding surface of TM6 with Gα_i/o_ of 5‐HT_1A_. TM6 beyond R^6.29^ was considered as extended helix. C) The additional contact areas of G_s_‐ or G_i/o_‐coupled receptors between the extended TM5/TM6 with Gα_s_/Gα_i/o_, respectively. D) Schematic representation of the TM5‐ (up) or TM6‐ (down) breaking mutants. E) Dose‐dependent curves for histamine induced cAMP accumulation or G‐protein dissociation in HEK293 cells expressing the truncated receptors (*n*  = 3). (Up) The TM5 of H_2_R was respectively truncated by four amino acids and eight amino acids. The cAMP assay validated that the length of TM5 was closely related to the activation of the G_s_ signal pathway. (Down) The TM6 of H_3_R was respectively truncated by eight amino acids and nine amino acids. The NanoBit assay validated that the length of TM6 did not correlate with the dissociation of the G_i_‐protein. F) Schematic representation of the geometry determinants for G‐protein selectivity of GPCRs.

Besides, with the aid of PCA visualization, we can depict a probable decision boundary given by our best classifier to derive a simpler formula (Figure 6F). The decision boundary elucidates that GPCRs with extended TM5 length, significant TM5 tilt, and prominent TM6 outward movement are indicative of G_s_‐coupling receptors. In contrast, GPCRs featuring shorter TM5 length, minimal TM5 tilt, and constrained TM6 outward movement are more likely associated with G_i/o_‐coupling receptors (**Figure** **7**F).

### 2.7 TM6 Length is not Significant for G_s_/G_i/o_ Selectivity

Structuromic analysis and ML analysis of GPCR‐G‐protein complexes suggested that the length of TM6 was not responsible for G_s_ and G_i/o_ selectivity. Therefore, further investigation is required to elucidate the mechanisms underlying the differential effects of TM5 and TM6 length in G‐protein selectivity. In the GPCR‐G_s_ complex, TM5 extends to insert into the groove formed by the αG‐α4 loop, the α4 helix, and the α4‐β6 loop of Gα_s_ (Figure 7A), leading to increased interactions with Gα_s_. To achieve this, extended TM5 tilts toward TM6 to fit into the groove in Gα_s_. Failure to accomplish this could result in severe steric hindrance with the αG‐α4 loop of Gα_s_, as observed in D1R (Figure 7A),^[^ ^28d^ ^]^ indicating a correlation between the length of TM5 and its tilt in the G_s_‐coupling receptors. Consistent with this, biplot analyses of GPCRdb‐predicted structures and true structures consistently reveal a high correlation between the length and tilt of TM5 in G_s_‐coupling receptors, with coefficients of 0.54 and 0.63, respectively (Figure S8, Supporting Information). However, the groove in Gα_s_ is absent in Gα_i/o_ due to the absence of 13 residues in the αG‐α4 loop (Figure 7B). Consequently, in the GPCR‐G_i/o_ complex, the extended TM5 and TM6 can only rest on the flattened surface of Gα_i/o_, resulting in smaller contact areas and fewer interactions compared to the G_s_‐coupled structure.

To validate this hypothesis, we investigated whether the extended TM5 in G_s_‐coupled structures or extended TM6 in G_i/o_‐coupled structures contributed equivalently to the increase in the contact area. Among the 54 true GPCR‐G structures, the median length of the entire TM5 is 23 in 20 G_s_‐coupled structures and 18 in 34 G_i/o_‐coupled structures (Table S5, Supporting Information). The median length of TM6 is 20.5 in G_s_‐coupled receptors and 22 in G_i/o_‐coupled structures. Consequently, we considered TM5 longer than 18 (below 5.69) in G_s_‐coupled receptors and TM6 longer than 20.5 (below 6.29) in G_i/o_‐coupled receptors as extended TMs. Interestingly, we found that the additional contact area between the extended TM5 and Gα_s_ exhibited a linear correlation with the extended residue amount, ≈40±3.0 Å^2^ per residue (R^2^ = 0.91) (Figure 7C; Figure S9 and Table S9, Supporting Information). For example, the contact area between the 15‐residue‐extended TM5 of D1R with Gα_s_ reached 561 Å^2^ (Figure 7A). In contrast, the increased contact area between TM6 and Gα_i/o_ did not show a significant correlation with the length of the extended TM6. For instance, the contact area between the 15‐residue‐extended TM6 of 5‐HT_1A_ and Gα_i_ was only 192.9 Å^2^ (Figure 7B).

Visualizing cryo‐EM density map of G_i/o_‐coupled complexes further revealed that the extended TM6 is not significant for G‐protein coupling. The densities for the extended TM6 in several G_i_‐coupled complex including 5‐HT_1A_, 5‐HT_1B_, and 5‐HT_1D_ are dramatically weaker and lack high‐resolution features compared with the rest of the receptor, suggesting the instable interactions between the extended TM6 and G_i_‐protein (Figure S10, Supporting Information).^[^ ^25^, ^29^ ^]^ Consistently, the removal of the extended TM6 of H_3_R posed negligible effect on its G_i_ activity even when nine residues were removed from the cytosolic extremity of TM6 (Figure 7D,E; Table S10, Supporting Information). In contrast, cAMP accumulation experiments confirmed that the removal of the extended TM5 of H_2_R significantly reduced its G_s_‐protein activity (Figure 7D,E; Table S10, Supporting Information). In conclusion, our findings indicate that a long TM5 is crucial for G_s_‐coupled receptors, whereas the TM6 length is not significant for G_s_ and G_i/o_ selectivity.

## 3 Discussion

The four histamine receptors serve essential roles in pathophysiological and signaling events, rendering them significant drug targets. In this study, we presented the structures of one H_2_R‐G_s_ and two H_3_R‐G_i_ complexes, offering profound insights into the orthosteric pocket of histamine receptors and G‐protein selectivity. Through our investigation of the amthamine‐bound H_2_R structure and mutagenesis studies, we revealed the discrepancy in the ligand pocket of H_1_R and H_2_R that contributed to H_2_R selectivity for the agonist amthamine. Notably, our findings uncovered a unique binding pose for endogenous histamine in H_3_R, deviating from other monoamine receptors, as the amino group interacts with E206^5.46^ in H_3_R instead of the conserved D114^3.32^ found in other aminergic receptors. This observation coincided with the behavior of the antipsychotic drug quetiapine, which exhibits high or modest affinities for many aminergic receptors with the exception of H_3_R and H_4_R.^[^ ^34^ ^]^ Importantly, the structural geometry differences observed in TM5/TM6 between H_2_R‐G_s_ and H_3_R‐G_i_ structures led us to propose the determinants of primary G_s_‐ and G_i/o_‐coupling selectivity.

GPCRs predominantly regulate downstream signaling through the engagement and activation of four major G‐protein subtypes. Among these four members, G_s_ and G_i/o_ are two principal signaling pathways, for about 86% GPCRs are annotated to couple with G_s_‐ or G_i/o_‐protein.^[^ ^2a^ ^]^ The selective coupling of GPCRs to the G‐proteins is crucial for effective signal transduction, especially those primarily coupled with G_s_‐ and G_i/o_‐proteins with remarkable coupling efficiency and rapid kinetics. The explosion of GPCR–G‐protein structures in recent years has significantly enhanced our comprehension on GPCR coupling mechanisms. Receptors and G‐proteins interact through a common mode, wherein the ligand‐activated GPCR opens its intracellular cavity and accommodates the α5 of Gα, which is conserved through the family/class A, B1, B2, and F GPCRs.^[^ ^9^, ^35^ ^]^ From the perspective of the G‐protein, the selectivity‐determining positions in Gα predominantly attribute to the α5 helix, along with the αN‐β1 loop, the β2‐β3 loop, and the α4 helix of Gα.^[^ ^1b^ ^]^ However, the selectivity signatures of receptors are more complicated due to the great divergence in the amino acid sequences and structural conformations of GPCRs. In contrast to the 16 members of Gα proteins which adopt similar conformations, the intracellular parts of GPCRs are more diverse, particularly in the regions of TM5, TM6, H8, intracellular loops, and the receptor C‐terminal tail. By combining reported GPCR–G‐protein structures with unpublished structures from our laboratory, we obtained a number of non‐redundant G_s_‐coupled or G_i/o_‐coupled structures of class A receptors, totaling 20 and 34, respectively. These structures revealed that the selectivity of G_s_ and G_i/o_ is determined by TM5/TM6 geometries, including the length and tilt of TM5, and the outward movement of TM6. The length and tilt of TM5 exhibit a high correlation in true GPCR‐G_s_ complexes, consistent with the extended and tilted TM5 (e.g., H_2_R) inserting into the groove formed by the αG‐α4 loop, the α4 helix, and the α4‐β6 loop of Gα_s_. However, the αG‐α4 loop is 13 residues shorter in Gα_i/o_ than in G_s_, which cannot constitute the same groove as Gα_s_ in Gα_i/o_. Hence, the extended TM6 (e.g., H_3_R) leads to less contact with Gα_i_ in G_i/o_‐coupled structures. This phenomenon indicates that the interactions of GPCR‐G_s_ are stronger than that of GPCR‐G_i/o_ complexes, which is consistent with Matic et al.**’**s report that the binding energy of G_s_ complexes is stronger than that of G_i/o_ complexes.^[^ ^13^ ^]^ Furthermore, our “TM5/6‐breaking” experiments also confirmed that TM6 length is not significant for G_s_ and G_i/o_ selectivity.

The intricate molecular mechanism underlying primary G‐protein coupling selectivity in GPCRs remains complex and not fully elucidated. The structures of GPCR–G‐protein complexes indicate that the ICL2, ICL3, TM5, and TM6 of receptors are associated with G‐protein‐coupling selectivity. However, these cytoplasmic regions of receptors rarely exhibit common patterns in terms of the sequence or amino‐acid properties related to G‐protein selectivity. In our prior study, we proposed that the TM5 and TM6 helices synergistically alternate lengths to determine the selectivity between G_s_‐ and G_i/o_‐proteins, defined as “a macro‐switch”.^[^ ^11^ ^]^ Comparative sequence analyses revealed that specific amino acids function as “micro‐switches” uniquely localized in the complementary pocket. However, these two switches only partially determine the selectivity between G_s_ and G_i/o_. Aided by the drastic increasing number and improved qualities of the structures of GPCRs in complexes with different G‐proteins in house and in the public data server, in the present study, we conducted a systematic evaluation to thoroughly explore the intricate relationship between the four geometries of TM5/TM6 and their impact on primary G‐protein selectivity by using machine learning‐based structuromic profiling. Using the GPCRdb homology models as the training dataset, we generated a G_s_‐ or G_i/o_‐coupling classifier that achieved an impressive 91% accuracy in the true dataset based on the TM5/TM6 geometries. Based on our classifier, we find that GPCRs with a long TM5 length and large TM5 tilt and TM6 outward movement are G_s_‐coupling receptors, whilst GPCRs with a short TM5 length and small TM5 tilt and TM6 outward movement are more likely to be G_i/o_‐coupling receptors. We anticipate that GPCRs that fail to satisfy the aforementioned conditions probably have comparable or promiscuous activity in stimulating G_s_ and G_i/o_ signaling (Figure 7F). Interestingly, class B1 receptors, primarily coupled to G_s_‐protein, exhibit large TM5 tilt and TM6 outward movement. These structural geometries also conform to the G‐protein selectivity features observed in Class A receptors, further demonstrating the universality of our proposed G‐protein selectivity mechanism (Table S11, Supporting Information). In summary, our studies revealed that the structural geometry of TM5/TM6 serves as the determinant of the primary G‐protein signaling selectivity in GPCRs. These findings have the potential to aid efforts in engineering GPCR selectivity, especially by modifying promiscuous GPCRs to improve affinity to specific G‐proteins.

## 4 Experimental Section

### Constructs

The wild‐type human H_2_R gene was cloned into the pFastBac1 vector. The N‐terminus of H_2_R was fused with the hemagglutinin signal peptide (HA) to enhance receptor expression, followed by a Flag tag (DYKDDDK) to facilitate complex purification. The wild‐type human H_3_R_445_ isoform was cloned into a modified pFastBac1 vector with a hemagglutinin (HA) signal sequence at the N‐terminus and a PreScission protease site followed by a Flag tag. The C‐terminus of H_3_R was fused with the LgBiT,^[^ ^22^ ^]^ followed by a TEV protease site and a double MBP tag to facilitate expression and purification. The dominant‐negative bovine Gα_s_ (DNGα_s_) and human Gα_i1_ (DNGα_i1_) were generated by site‐directed mutagenesis, as previously described, to stabilize the interaction with the βγ subunits,^[^ ^36^ ^]^ and were cloned into the pFastBac1 vector. Human Gβ1 was fused with a 10× His tag at the N‐terminus and HiBiT at the C‐terminus, and together with Gγ2, was cloned into the pFastBac dual vector.

### Protein Complex Expression and Purification

For the H_2_R‐G_s_ complex, the H_2_R, DNGα_s_, and Gβ1γ2 constructs were co‐expressed in Spodoptera frugiperda (Sf9) cells using the Bac‐to‐Bac Baculovirus Expression System (Invitrogen). Cells were infected at a density of 2.4 × 10^6^ cells per mL and then co‐infected with three separate viruses at a ratio of 2:1:1 for H_2_R, DNGα_s_, and Gβ1γ2. Cells were collected 48 hours post‐infection and stored at −80 °C until use. Sf9 cell pellets expressing H_2_R and G‐protein trimer were resuspended in a buffer containing 20 mm HEPES pH 7.4, 100 mm NaCl, 3 mm MgCl_2_, 5 mm CaCl_2_, 2.5 mg mL^−1^ leupeptin, and 0.2 mg mL^−1^ benzamidine. The complex was assembled on the membrane by adding 100 µm amthamine, 10 mg mL^−1^ Nb35, 25 mU mL^−1^ apyrase, and further incubated at RT for 2 h. The complex was then extracted from the cell membrane using 0.5% (w/v) lauryl maltose neopentylglycol (LMNG) (Anatrace) and 0.1% (w/v) cholesteryl hemisuccinate (CHS) (Anatrace) at 4 °C for 2 h. After that, the supernatant was collected by centrifugation at 25 000 rpm for 30 min, and the solubilized complex was incubated with M1 anti‐FLAG resin for 2.5 h at 4 °C. Afterwards, the resin was collected and washed with a buffer containing 20 mm HEPES pH 7.4, 100 mm NaCl, 3 mm MgCl_2_, 5 mM CaCl_2_, 0.1% (w/v) LMNG, 0.02% (w/v) CHS, 100 µM amthamine, 2.5 mg mL^−1^ leupeptin, and 0.2 mg mL^−1^ benzamidine. Subsequently, the detergent was changed to 0.01% (w/v) LMNG, 0.005% (w/v) CHS. Then, the H_2_R‐G_s_ complex was eluted with 20 mm HEPES pH 7.4, 100 mm NaCl, 0.01% (w/v) LMNG, 0.001% (w/v) CHS, 5 mm EGTA, 0.2 mg mL^−1^ FLAG peptide, 100 µM amthamine, 2.5 mg mL^−1^ leupeptin, and 0.2 mg mL^−1^ benzamidine. The eluate was collected and injected onto a Superdex 6 Increase 10/300 GL column (GE Healthcare) with buffer containing 20 mm HEPES pH 7.4, 100 mm NaCl, 0.00025% (w/v) LMNG, 0.0002% (w/v) CHS, 0.00075% (w/v) GDN (Anatrace), and 100 µm amthamine. The complex fractions were concentrated with a 100 kDa MWCO Millipore concentrator to 8 mg mL^−1^ for making a cryo‐EM grid.

For the H_3_R‐G_i_ complex, the H_3_R_445_ isoform, DNGα_i1_, and Gβ1γ2 constructs were co‐expressed in Spodoptera frugiperda (Sf9) cells using the Bac‐to‐Bac Baculovirus Expression System (Invitrogen). Cells were infected at a density of 2.4 × 106 cells per mL and then co‐infected with three separate viruses at a ratio of 1:1:1 for H_3_R, DNGα_i1_, and Gβ1γ2. Cells were collected 48 h post‐infection and stored at −80 °C until use. The co‐expressed H_3_R‐G_i_ complex cell pellets were lysed in a buffer containing 20 mm HEPES pH 7.5, 100 mm NaCl, 2 mm MgCl_2_, adequate agonist (1 mm histamine or 10 µm immepip), and supplemented with EDTA‐free protease inhibitor cocktail (Bimake) using a Dounce homogenizer. The complex formation was initiated by adding 20 µg mL^−1^ scFv16 and 25mU mL^−1^ apyrase (NEB) and subsequently incubated for 1.5 h at RT. Cell membranes were solubilized by 1% (w/v) n‐Dodecyl‐β‐D‐Maltopyranoside (DDM, Anatrace) and 0.2% (w/v) cholesterol hemisuccinate (CHS, Anatrace) for 2.5 h at 4 °C. After centrifugation at 30 000 g for 30 min, the supernatant was isolated and incubated with amylose resin (NEB) for 1 hours at 4 °C. Then, the resin was washed with a wash buffer containing 20 mm HEPES pH 7.5, 100 mm NaCl, 2 mm MgCl_2_, 0.1% (w/v) LMNG, 0.02% (w/v) CHS, and agonist (1 mm histamine or 10 µm immepip). Subsequently, the detergent was changed to 0.01% (w/v) LMNG, 0.005% (w/v) CHS, and eluted with an elution buffer containing 20 mm HEPES pH 7.5, 100 mm NaCl, 2 mm MgCl_2_, 0.01% (w/v) LMNG, 0.005% (w/v) CHS, 10 mm Maltose, and agonist (1 mm histamine or 10 µm immepip). The double MBP tag of H_3_R was removed by incubating with TEV protease for 1 hour at RT. Then, the protein sample was further purified by size‐exclusion chromatography with a Superose 6 10/300 GL column (GE Healthcare) equilibrated with a running buffer containing 20 mm HEPES pH 7.5, 100 mm NaCl, 2 mm MgCl_2_, 0.00225% (w/v) LMNG, 0.0006% (w/v) CHS, 0.00075% (w/v) GDN (Anatrace), and agonist (1 mM histamine or 10 µM immepip).

### Cryo‐Grid Preparation and EM Data Collection

Three microliters of the purified GPCR–G‐protein complexes were applied onto a glow‐discharged holey carbon grid (Quantifoil R1.2/1.3) at ≈20 mg mL^−1^. The grids were plunge‐frozen in liquid ethane using Vitrobot Mark IV (Thermo Fisher Scientific). The frozen grids were then transferred to liquid nitrogen and stored for data collection.

Cryo‐EM imaging was performed on a Titan Krios at 300 kV using the Gatan K2 Summit detector at the Center of Cryo‐Electron Microscopy, Zhejiang University (Hangzhou, China). Movies were recorded at a dose rate of approximately 8.0 e/Å2/s with a defocus ranging from −1.0 to −2.2 µm using the SerialEM software in counting mode for the three receptor‐G‐protein complexes, respectively. The total exposure time was 8 seconds and 40 frames were recorded per micrograph. A total of 3122, 3008, and 3079 movies were collected for the amthamine‐H_2_R‐G_s_, histamine‐H_3_R‐G_i_, and immepip‐H_3_R‐G_i_ complexes, respectively, for structure reconstruction.

### Image Processing and 3D Reconstructions

Image stacks were aligned using MotionCor 2.1.^[^ ^37^ ^]^ Contrast transfer function (CTF) parameters were estimated using Gctf v1.18.^[^ ^38^ ^]^ The following data processing was performed using RELION‐3.0‐beta2.

For the amthamine‐H_2_R‐G_s_ complex, automated particle picking yielded 3361930 particles. The particles extracted from the dataset were downscaled two times and subjected to 2D classification. The map of the GPBAR‐G_s_ complex (EMD‐30344),^[^ ^14c^ ^]^ low‐pass filtered to 40 Å, was used as an initial reference model for two rounds of 3D classification, resulting in a well‐defined subset of 857906 particles. The particles were then re‐extracted with the original pixel size and performed further 3D classification focusing on the receptor‐G_s_ complex, which produced a good subset accounting for 368447 particles. After 3D refinement, CTF refinement, and Bayesian polishing of the final particles, the final refinement map was generated with a global resolution of 2.7 Å at a Fourier shell correlation of 0.143.

For the histamine‐H_3_R‐G_i_ complex, template‐based particle selection produced 2487329 particles. After 2D classification and two rounds of 3D classification using the 5‐HT_1D_‐G_i_ complex (EMD‐30974)^[^ ^25a^ ^]^ low‐pass filtered map as an initial reference model, a well‐defined subset with 742110 particles was selected. Further 3D classifications focusing on the alignment of the receptor‐G_i_ complex produced a good subset accounting for 234203 particles, which were subsequently subjected to 3D refinement, CTF refinement, and Bayesian polishing. The final refinement generated a map with an indicated global resolution of 2.7 Å at a Fourier shell correlation of 0.143.

For the immepip‐H_3_R‐G_i_ complex, template‐based particle selection produced 2050983 particles. After 2D classification and two rounds of 3D classification using the histamine‐H_3_R‐G_i_ complex low‐pass filtered map as an initial reference model, two well‐defined subsets with 697323 particles were selected. Further 3D classifications focusing on alignment of the receptor produced a good subset accounting for 330449 particles, which were subsequently subjected to 3D refinement, CTF refinement, and Bayesian polishing. The final refinement generated a map with an indicated global resolution of 3.0 Å at a Fourier shell correlation of 0.143.

### Model Building and Refinement

For the H_2_R‐G_s_ complexes, the initial model of H_2_R was downloaded from the activated homology models of H_2_R from GPCRdb.^[^ ^31^ ^]^ The initial G_s_ and Nb35 complex was generated from the GPBAR‐G_s_ complex (PDB ID: 7CFM).^[^ ^14c^ ^]^ For the H_3_R‐G_i_ complex, the initial model of H_3_R was downloaded from GPCRdb as H_2_R. The initial G_i_ and scFv16 complex was generated from the 5‐HT_1D_‐G_i_ complex (PDB ID: 7E32).^[^ ^25a^ ^]^ Agonist and lipid coordinates and geometry restraints were generated using phenix.elbow. Then, the models were docked into the cryo‐EM density map using Chimera. After the initial docked models were refined using Rosetta, the models were subjected to iterative rounds of manual adjustment and auto‐refinement in Coot and Phenix, respectively. The final refinement scores were validated by the module “comprehensive validation (cryo‐EM)” in Phenix. Structure figures were prepared using PyMOL, Chimera, and ChimeraX.

### Molecular Dynamics Simulations

First, the histamine bound to H_3_R model was subtracted from histamine‐H_3_R‐G_i_ complex. Second, the histamine was also positioned in a similar direction to the histamine‐H_1_R structure and fitted to the cryo‐EM density. The orientations of the receptors were calculated by the Positioning of Proteins in Membranes (PPM) Web Server.^[^ ^39^ ^]^ Following these steps, the whole systems were prepared using the CHARMM‐GUI and embedded in a bilayer consisting of 200 1‐palmitoyl‐2‐oleoyl‐sn‐glycero‐3‐phosphocholine (POPC) lipids using replacement methods.^[^ ^40^ ^]^ The membrane systems were then solvated into a periodic TIP3P water box supplemented with 0.15 m NaCl. The CHARMM36m Force Field was used to model protein molecules, and the CHARMM General Force Field (CGenFF) was used for the agonist histamine.

Subsequently, two systems were subjected to minimization for 10 000 steps using the conjugate gradient algorithm. They were then heated and equilibrated at 310.13 K and 1 atm for 200 ps with 10.0 kcal mol^−1^ Å^−2^ harmonic restraints in the NAMD 2.13 software. This was followed by 5 cycles of equilibration for 2 ns each at 310.13 K and 1 atm, during which the harmonic restraints were sequentially reduced to 5.0, 2.5, 1.0, 0.5, 0.1 kcal mol^−1^ Å^−2^. Production simulations were run at 310.13 K and 1 atm in the NPT ensemble using the Langevin thermostat and Nose‐Hoover method for 250 ns. Electrostatic interactions were calculated using the particle mesh Ewald (PME) method with a cutoff of 12 Å. Throughout the final stages of equilibration and production, 5.0 kcal mol^−1^ Å^−2^ harmonic restraints were placed on the backbone of the receptors due to the flexibility of the second extracellular loop (ICL2) of H_3_R. Trajectories were visualized and analyzed using Visual Molecular Dynamics (VMD) version 1.9.3.

### GloSensor cAMP Assay

The agonist‐induced cAMP accumulation was measured using the GloSensor™ cAMP assay kit (Promega). HEK293T cells were co‐transfected with a 3:1 ratio of WT or mutant and the pGloSensor™−22F plasmid by using LIPO (YEASON). After a minimum of 6 h, transfected cells were seeded onto cell adherent reagent (Applygen) coated 384‐well culture plates and incubated for more than 12 h at 37 °C in 5% CO_2_. The culture medium was removed and PBS was added to starve the cells. After 20 min later, the PBS was removed, and the cells were incubated with the equilibration medium (CO_2_‐Independent Medium (Gibco) with 10% FBS) containing a 4% dilution of the GloSensor™ cAMP reagent stock solution for 30 minutes at 37 °C and then 10 min at RT. To obtain the dose‐response curves, serially diluted agonists were added to each well to stimulate the cells. Luminance signal was measured using 200 ms intervals then (TECAN, 25 °C). Dose‐responses were generated from the peak response. cAMP accumulation was analyzed by a standard dose‐response curve using GraphPad Prism 8.0 (GraphPad Software). EC50 and pEC50± SEM were calculated using nonlinear regression (curve fit). Data are means ± SEM from at least three independent experiments performed in technical triplicates.

### NanoLuc Binary Technology (NanoBiT) Assay

For the measurement of G‐protein dissociation, HEK293T cells were co‐transfected with WT or mutant, Gα_i_‐LgBit, Gβ, and Gγ‐SmBit plasmids at a 6:2:5:5 ratio. Following transfection, cells were seeded onto 96‐well culture plates and incubated for over 12 h at 37 °C in 5% CO_2_. Then, the cells were rinsed twice with D‐PBS and incubated with 4 nm coelenterazine‐400a (Maokangbio) in HBSS supplemented with 5 mm HEPES pH 7.4 and 0.1% BSA for 1 h. The baseline luminance signal was read immediately for 5 cycles (TECAN, 25 °C). To obtain the dose‐response curves, serially diluted agonists were added to each well to stimulate the cells. The luminance signal was then measured for another 15 cycles using 500 ms intervals (TECAN, 25 °C). Dose‐responses were generated from the 5th response, and the row data was standardized by the baseline and the vehicle group. G‐protein activation was analyzed by a standard dose‐response curve using GraphPad Prism 8.0 (GraphPad Software). EC50 and pEC50± SEM were calculated using nonlinear regression (curve fit). Data are means ± SEM from at least three independent experiments performed in technical triplicates.

### Enzyme Linked Immunosorbent (ELISA) assay

To confirm the cell surface expression of H_3_R and its mutants, the ELISA was performed 24 h after transfection using cells plated on 96‐white plates. The cells were fixed with 4% formaldehyde for five minutes at RT and then washed once with PBS. Following fixation, the cells were blocked with blocking buffer (1% FBS in PBS) for 2 h at RT. Afterwards, the plates were incubated with a 1:10000 dilution of monoclonal ANTI‐FLAG M2‐Peroxidase (HRP) antibody (Sigma Aldrich) in blocking buffer for another 0.5 h at RT. After careful washing, 80 µL per well of SuperSignal ELISA Femto Maximum Sensitivity Substrate (ThermoFisher Scientific) solution was added. The luminance signal was measured using 500 ms intervals. The data are presented as means ± SEM from at least three independent experiments performed in technical triplicates.

### Collections of Activated GPCR Data Sets

For the true GPCR datasets, structures were collected from reported distinct class A GPCR–G‐protein complex structures that are primarily coupled to G_s_‐ or G_i/o_‐proteins. Several G_s_‐ or G_i/o_‐coupled receptors that are primarily coupled to G_q_ protein, such as CCK_1_R, CCK_2_R, GASR, NK_1_R, and NTSR1, were excluded. The true datasets were enlarged to 20 G_s_‐ and 34 G_i/o_‐coupled structures by adding the four G_s_‐coupled and one G_i_‐coupled complexes from our unpublished data.

For the GPCRdb homology model datasets, the primary G_s_‐ or G_i/o_‐coupled activated class A GPCR homology models were collected from GPCRdb. However, the accuracy of orphan class A receptors homology models is poor due to the lack of true homologous structures. After removing the orphan receptors, 98 (24 G_s_‐ and 74 G_i/o_‐coupled receptors) GPCRdb homology models were used as training datasets.

### Extractions of Length and Angle

To conveniently extract the length and angle geometries of receptors, the reference H_3_R model was positioned on the lipid bilayer using the PPM server. The 7TMs of H_3_R were perpendicular to the membrane plane. Then, the structures of true GPCR dataset and GPCRdb homology model dataset were superimposed onto H_3_R. The Z‐axis of all receptor models was perpendicular to the membrane plane. The lengths of TM5 and TM6 were measured from the cytosolic tip to 5.50 and 6.50, respectively. The angle of TM5 and TM6 was calculated between the orientation of a helix and the reference direction. The orientation of a helix was obtained by averaging the C = O bond orientation of each amino acid on the helix segment. Considering that an alpha helix turn consists of 3.6 amino acids, we selected 11 amino acids from the cytoplasmic tip of TM5 or TM6 to calculate the average orientation and eliminate the component perpendicular to the helix axis. The reference orientation for TM5 angle calculation is the TM5 orientation of H_3_R, in which the average C = O bond orientation is 22 amino acids instead of 11. As the TM6 angle indicates the TM6 outward movement during receptor activation, the reference orientation for the TM6 angle is the central axis of the receptors, which is the Z‐axis that is perpendicular to the membrane plane.

### Data Pre‐Processing and Machine Learning Classification

We utilize the Sklearn package^[^ ^41^ ^]^ to perform the data pre‐processing and machine learning tasks. First, the TM6 length is excluded due to the statistical reason previously stated, followed by standardizing, namely, centering and scaling the three‐geometries dataset. From above, we construct a training set of (3,n) dimensions where *n* = 98 (Gs:24, Gi:74) is the number of data points in the training set.

For the cross‐validation, the stratified 10‐fold validation is used on the GPCRdb set. After that, we train the Random Forest classifier on the whole training set with the parameters as follows (unspecified parameters set as default): RandomForestClassifier(max_depth = 1, n_estimators = 15, max_features = 1, class_weight = {0:74, 1:24}), in which the bagging strategy is used with sample ratio as 0.7. Note that the standardization fit with the training dataset is applied to the true dataset as well. The best model is trained with random seed as 2784 for both bagging and Random Forest.

Subsequently, we apply PCA to fit the training dataset and get a PCA transformation for visualization. The technique of linear dimensionality reduction, known as Principal Component Analysis (PCA), employs the Singular Value Decomposition method to project the data into a subspace of reduced dimensions. We use the PCA implementation in sklearn.decomposition and it can be used with the default parameters. The first two principal components are reserved, which explain over 87% variance of the data.

The resultant principle can be distilled from the decision boundary for approximate categorization. The comprehensive transformation equation transitioning from the raw domain to Principal Component (PC) space is as follows:

X = 0.649*1/4.164*(tm5_len – 20.276) + 0.569*1/5.264*(tm5_tilt – 8.474) + 0.506*1/9.156*(tm6_outward‐ 18.749)

Y = −0.081*1/4.164*(tm5_len – 20.276) + −0.609*1/5.264*(tm5_tilt – 8.474) + 0.789*1/9.156*(tm6_outward‐ 18.749)

Upon abstracting standardization, we obtain:

X = 0.649*tm5_len_std+0.569*tm5_tilt _std+0.506*tm6_outward_std,

Y = 0.081*tm5_len_std+ 0.609*tm5_tilt _std −0.789*tm6_outward_std

Herein, [0.649 0.569 0.506] and [−0.081 −0.609 0.789] are the corresponding eigenvectors for PC1 and PC2.

The following formula facilitates the direct calculation of the corresponding values:

X = 0.156*tm5_len + 0.108*tm5_tilt + 0.055*tm6_outward – 5.110

Y = −0.020*tm5_len – 0.116*tm5_tilt + 0.086*tm6_outward – 0.239

When X>0.7, it is highly probable that GPCR will couple with G_s_, indicative of an elongated TM length, a substantial TM tilt, and a significant outward shift in TM6 orientation. Conversely when X falls below or equals 0.7, there's an increased likelihood of GPCR coupling with G_i_ which comprises instances that are either Y.

### Statistical Analysis

Data were represented as the mean ± SEM values of ≥3 independent experiments. The raw data of the NanoBiT assay was standardized by the baseline and the vehicle group, while the raw data of cAMP and ELISA assay was standardized by the corresponding WT group. The sigmoidal curves of the reporter luciferase assay were analyzed using a standard dose‐response curve in GraphPad Prism version 8.02 (GraphPad Software, San Diego, CA, USA). Data sets with two groups were analyzed by Student's t‐tests. Data with ≥3 groups were analyzed by one‐way analysis of variance (ANOVA), while the violin plots depicting four distinct features of TM5/TM6 in G_s_‐ and G_i/o_‐coupled receptors are calculated using Mann‐Whitney U test taking advantage of scikit learn python package. A significance level of *p* < 0.05 was considered, and the details are provided in the figure legends.

## Conflict of Interest

The authors declare no conflict of interest.

## Author Contributions

Q.S., X.T., X.W., and S.C. contributed equally to this work. Y.Z. initiated and conceived the study. Y.Z., Z.C., J.‐P.S., W.H., and M.Z. supervised the project. Q.S. designed the expression constructs, optimized the sample purification, and prepared the final samples for cryo‐EM studies for the H_3_R‐G_i_ complexes. X.W., P.X., and J.‐P.S. designed the expression constructs and prepared the final samples for cryo‐EM studies for the H_2_R‐G_s_ complex. C.M., D.‐D.S., and H.Z. evaluated the specimen by negative‐stain EM, screened the cryo‐EM conditions, prepared the cryo‐EM grids, and collected cryo‐EM images. Q.S. performed density map calculations, model building, refinement of final models. Y.Z. and Q.S. analyzed the structure, designed the mutations, and functional assays. X.T., X.W., P.X., H.X., L.J., and Y.Z. performed the functional assays. M.Z., Y.Z., S.C., and Q.S. performed machine learning analyses. Q.S. and S.‐K.Z. performed molecular dynamics simulations. Q.S., X.T., S.C., and X.W. prepared the figures. Y.Z. and Q.S. wrote the manuscript. Z.C., J.‐P.S., W.H., and M.Z. provided important discussions and essential revisions.

## Supporting information

Supporting Information

## Data Availability

The Electron Microscopy Data Bank accession numbers and Protein Data Bank identifiers are EMD‐39582 and PDB ID 8YUT for the amthamine‐H_2_R‐G_s_ complex, EMD‐39583 and PDB ID 8YUU for the histamine‐H_3_R‐G_i_ complex, and EMD‐39584 and PDB ID 8YUV for the immepip‐H_3_R‐G_i_ complex, respectively. Other data that support the findings of this study are available in the supplementary material of this article.
