# Correction to “Molecular Determinant Underlying Selective Coupling of Primary G‐Protein by Class A GPCRs”

**DOI:** 10.1002/advs.202416290

**Published:** 2025-01-21

Shen Q, Tang X, Wen X, et al. Molecular Determinant Underlying Selective Coupling of Primary G‐Protein by Class A GPCRs. *Adv Sci (Weinh)*. 2024;11(23)

https://doi.org/10.1002/advs.202310120

In the first affiliation, the text:

Q. Shen, S. Cheng, S. Zang, D. Shen, H. Zhang, H. Xu, C. Mao, Y. Zhang

Department of Pharmacology and Department of Pathology of Sir Run Run Shaw Hospital & Liangzhu Laboratory, Hangzhou 310058, China

E‐mail: zhang_yan@zju.edu.cn

 was incorrect. This should have read:

Q. Shen, S. Cheng, S. Zang, D. Shen, H. Zhang, H. Xu, C. Mao, Y. Zhang

Department of Pathology of Sir Run Run Shaw Hospital, Department of Pharmacology, and Liangzhu Laboratory, Zhejiang University School of Medicine, Hangzhou 310058, China

E‐mail: zhang_yan@zju.edu.cn

Additionally, in the “Acknowledgements” Section, the text “the Ministry of Science and Technology (2019YFA050880 to Y.Z.)” was incorrect. This should have read: “The Ministry of Science and Technology (2019YFA0508800 to Y.Z.).”

We apologize for this error.

