# Supplementary material for: Molecular Determinant Underlying Selective Coupling of Primary G‐Protein by Class A GPCRs
Source: Adv Sci (Weinh). 2024 Apr 22;11(23):2310120. doi: 10.1002/advs.202310120 (PMC11187927; doi:10.1002/advs.202310120)
Supplement: Supplementary file 1 — Supporting Information [file ADVS-11-2310120-s001.pdf]

ADVANCED SCIENCE
Open Access

Supporting Information

for *Adv. Sci.*, DOI 10.1002/advs.202310120

Molecular Determinant Underlying Selective Coupling of Primary G-Protein by Class A GPCRs

Qingya Shen, Xinyan Tang, Xin Wen, Shizhuo Cheng, Peng Xiao, Shao-Kun Zang, Dan-Dan Shen, Lei Jiang, Yanrong Zheng, Huibing Zhang, Haomang Xu, Chunyou Mao, Min Zhang, Weiwei Hu*, Jin-Peng Sun*, Yan Zhang* and Zhong Chen**

Molecular Determinant Underlying Selective Coupling of Primary G-protein by Class A GPCRs

Qingya Shen[#], Xinyan Tang[#], Xin Wen[#], Shizhuo Cheng[#], Peng Xiao, Shao-Kun Zang, Dan-Dan Shen, Lei Jiang, Yanrong Zheng, Huibing Zhang, Haomang Xu, Chunyou Mao, Min Zhang, Weiwei Hu*, Jin-Peng Sun*, Yan Zhang*, and Zhong Chen**

Supporting Information

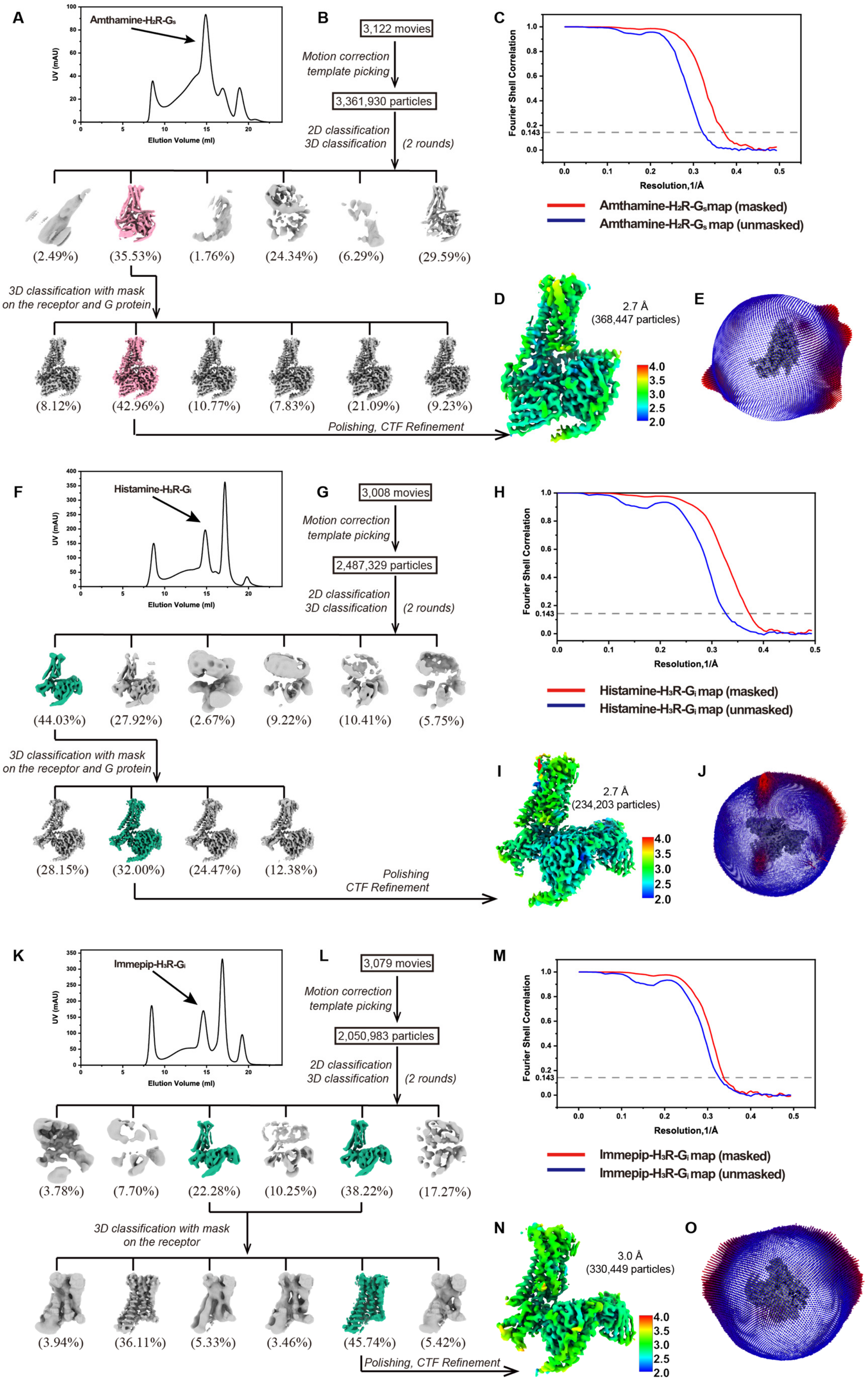

A
Amthamine-H2R-Gs
UV (mAU)
Elution Volume (ml)
B
3,122 movies
Motion correction
template picking
3,361,930 particles
2D classification
3D classification
(2 rounds)
(2.49%)
(35.53%)
(1.76%)
(24.34%)
(6.29%)
(29.59%)
3D classification with mask
on the receptor and G protein
(8.12%)
(42.96%)
(10.77%)
(7.83%)
(21.09%)
(9.23%)
Polishing, CTF Refinement
C
Fourier Shell Correlation
Resolution,1/Å
Amthamine-H2R-Gs map (masked)
Amthamine-H2R-Gs map (unmasked)
D
2.7 Å
(368,447 particles)
E
F
Histamine-H3R-Gi
UV (mAU)
Elution Volume (ml)
G
3,008 movies
Motion correction
template picking
2,487,329 particles
2D classification
3D classification
(2 rounds)
(44.03%)
(27.92%)
(2.67%)
(9.22%)
(10.41%)
(5.75%)
3D classification with mask
on the receptor and G protein
(28.15%)
(32.00%)
(24.47%)
(12.38%)
Polishing
CTF Refinement
H
Fourier Shell Correlation
Resolution,1/Å
Histamine-H3R-Gi map (masked)
Histamine-H3R-Gi map (unmasked)
I
2.7 Å
(234,203 particles)
J
K
Immepip-H3R-Gi
UV (mAU)
Elution Volume (ml)
L
3,079 movies
Motion correction
template picking
2,050,983 particles
2D classification
3D classification
(2 rounds)
(3.78%)
(7.70%)
(22.28%)
(10.25%)
(38.22%)
(17.27%)
3D classification with mask
on the receptor
(3.94%)
(36.11%)
(5.33%)
(3.46%)
(45.74%)
(5.42%)
Polishing, CTF Refinement
M
Fourier Shell Correlation
Resolution,1/Å
Immepip-H3R-Gi map (masked)
Immepip-H3R-Gi map (unmasked)
N
3.0 Å
(330,449 particles)
O

Figure S1. Preparation of amthamine-bound H_2R-G_s and histamine- and immepip-bound H_3R-G_i complexes and cryo-EM data processing. A-E) Purification and data processing of amthamine-bound H_2R-G_s complex. (A) Size exclusion chromatography (SEC) profile of the complex. (B) Flow chart of the cryo-EM data processing for the complex. (C) The "Gold-standard" Fourier shell correlation (FSC) curves of the complex. (D) Cryo-EM map of the complex colored by local resolution (Å). (E) Angular distribution map of the complex. F-J, Purification and data processing of histamine-bound H_3R-G_i complex. K-O, Purification and data processing of immepip-bound H_3R-G_i complex.

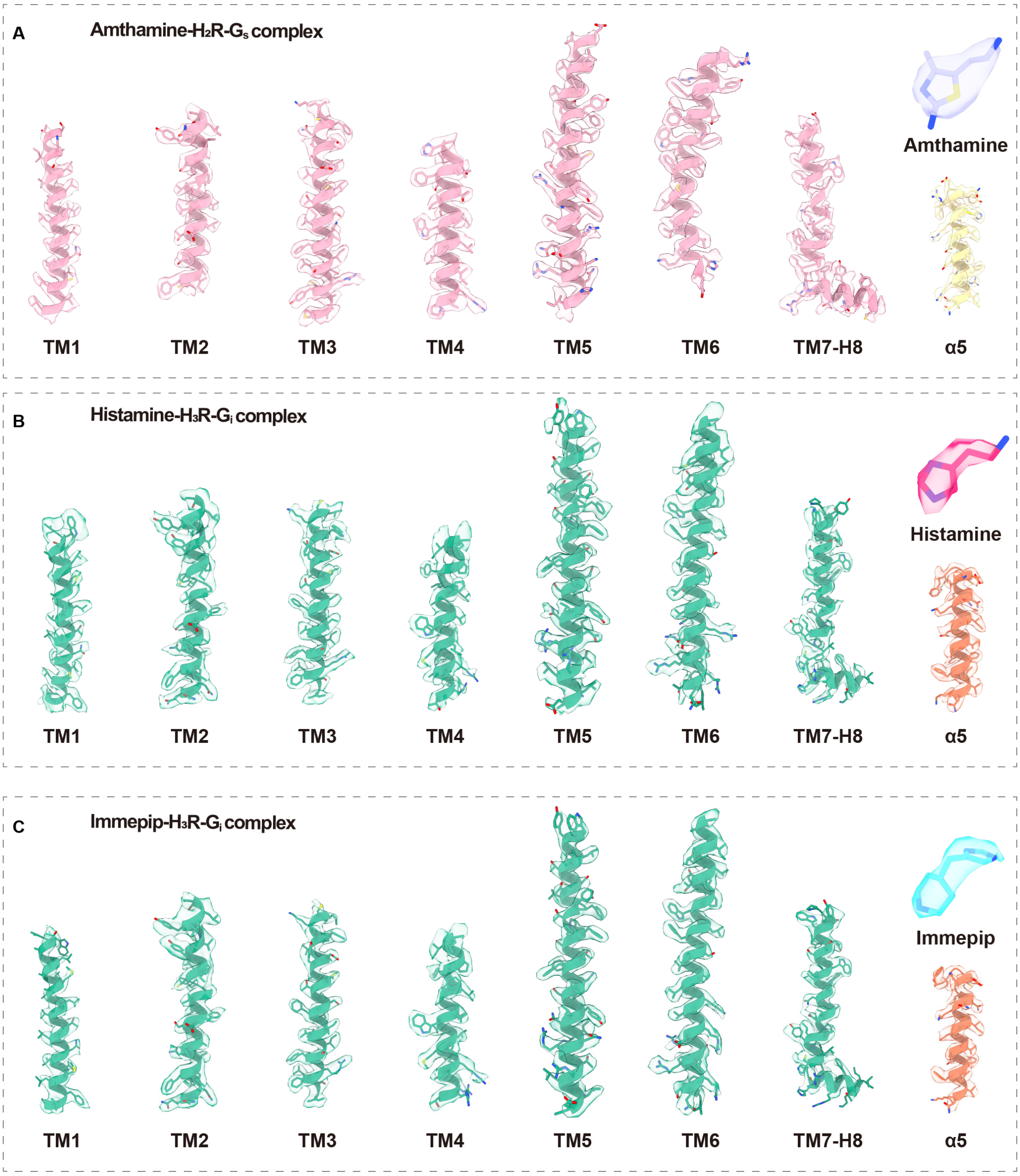

Figure S2. Cryo-EM quality of amthamine-bound H_2R-G_s and histamine- and immepip-bound H_3R-G_i complexes. A-C) EM density maps and models of 7TM of the receptor, the α5 helix of Gα, and agonist of amthamine-bound H_2R-G_s (A), histamine-bound H_3R-G_i (B), and immepip-bound H_3R-G_i (C) complexes.

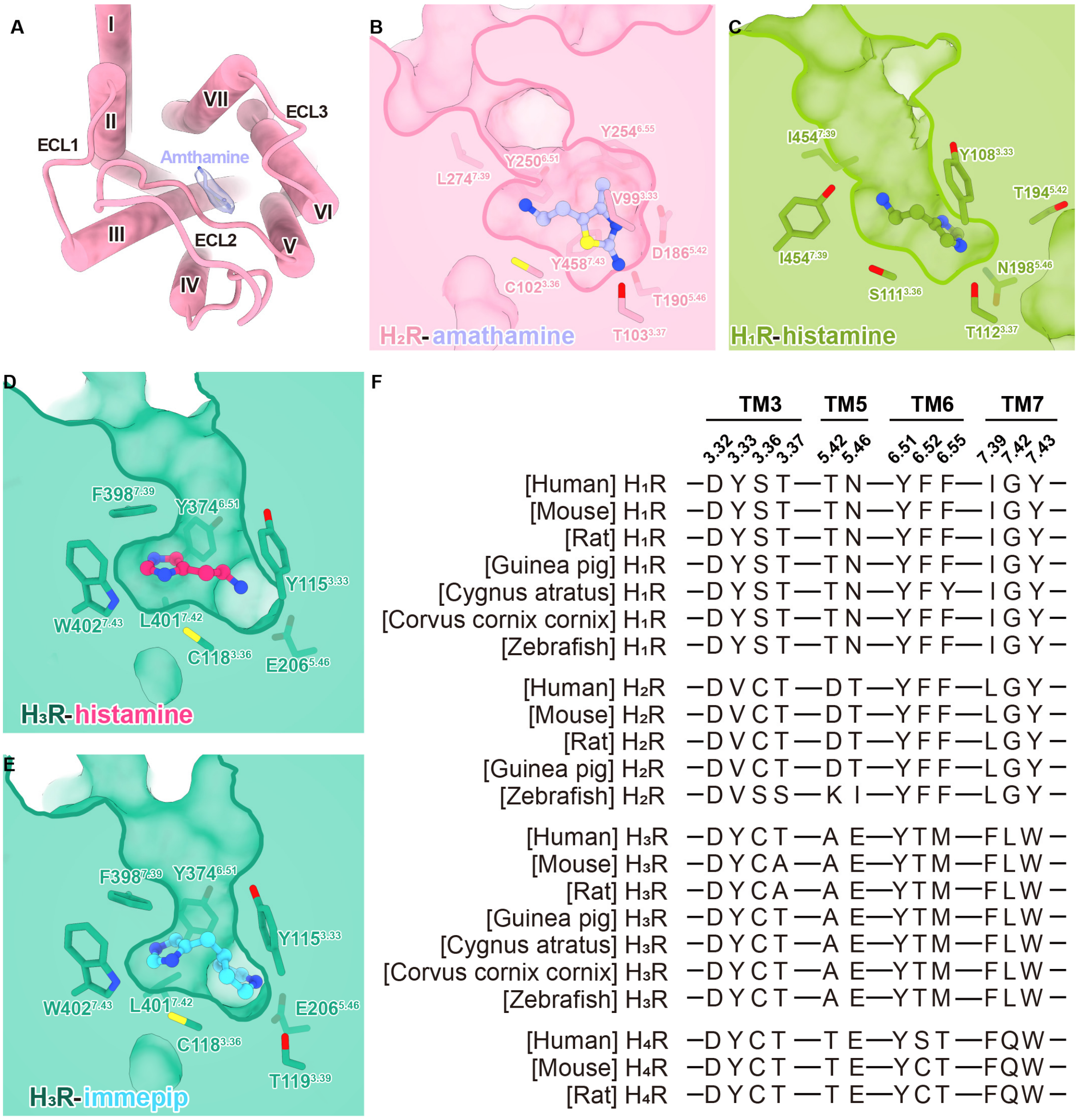

Figure S3. Ligand pocket of histamine receptors. A) Cartoon representation of the orthosteric pocket of H_2R. The cryo-EM density map of the ligand amthamine is displayed and colored in blue-violet. B-E) Sliced surface representations of the ligand pockets of the H_2R-amthamine (B), H_1R-histamine (C), H_3R-histamine (D), and H_3R-immepip (E) complexes. The receptors are presented as surfaces, while the residues in the ligand pocket are depicted as sticks. The ligands in the pockets are illustrated as ball-and-stick models. F) Sequence alignment of the orthosteric pocket of histamine receptors across different species.

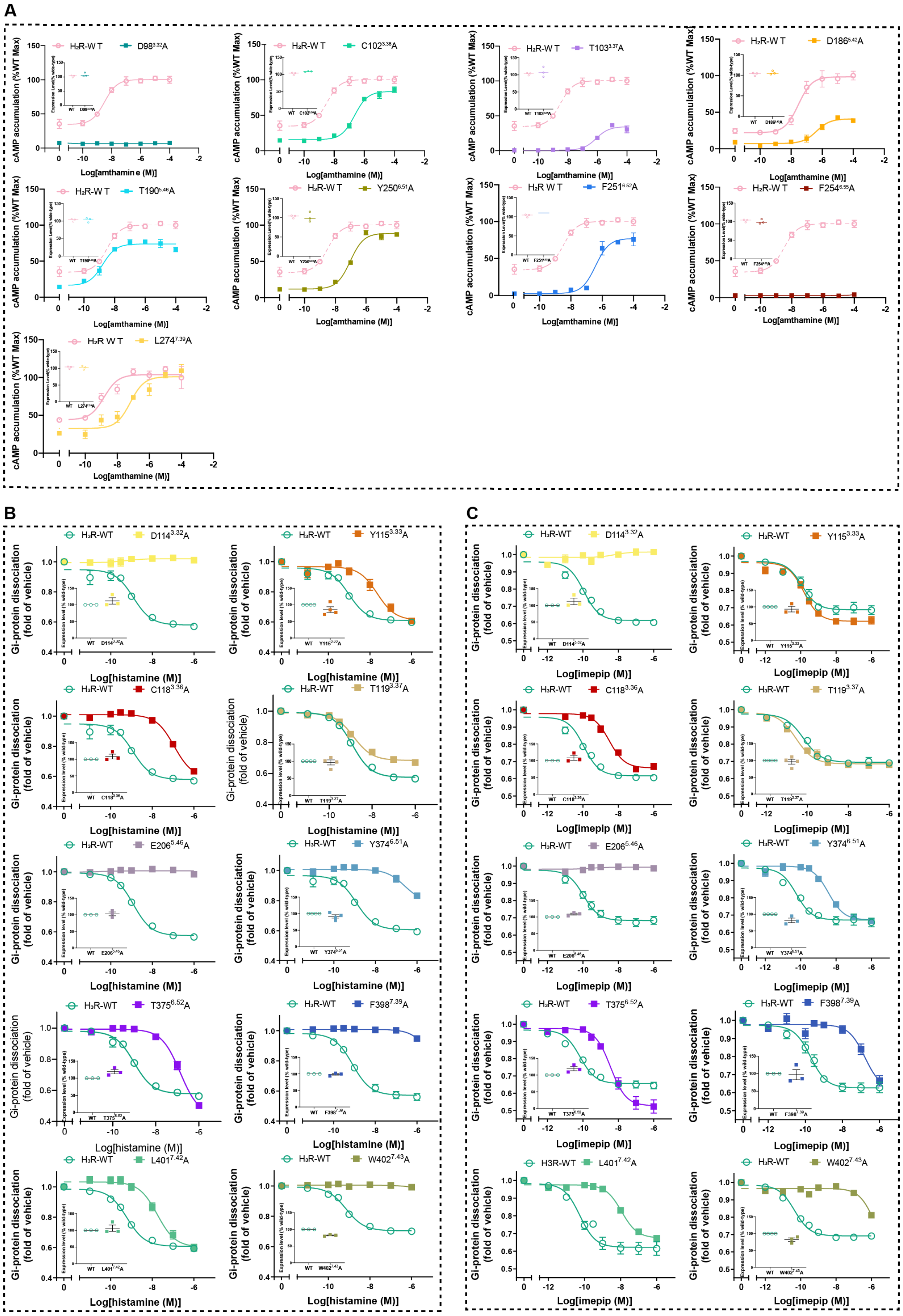

Figure S4. Functional analysis of H_2R and H_3R mutants. A) Dose-dependent curves for amthamine induced cAMP accumulation in HEK293 cells expressing the H_2R mutants of the residues in orthosteric pocket by GloSensor assays (n=3). B) Dose-dependent

curves for histamine induced G-protein dissociation in HEK293 cells expressing the H_3R mutants of the residues in orthosteric pocket by NanoBiT assays (n=3). C) Dose-dependent curves for immepip induced G-protein dissociation in HEK293 cells expressing the H_3R mutants of the residues in orthosteric pocket by NanoBiT assays (n=3).

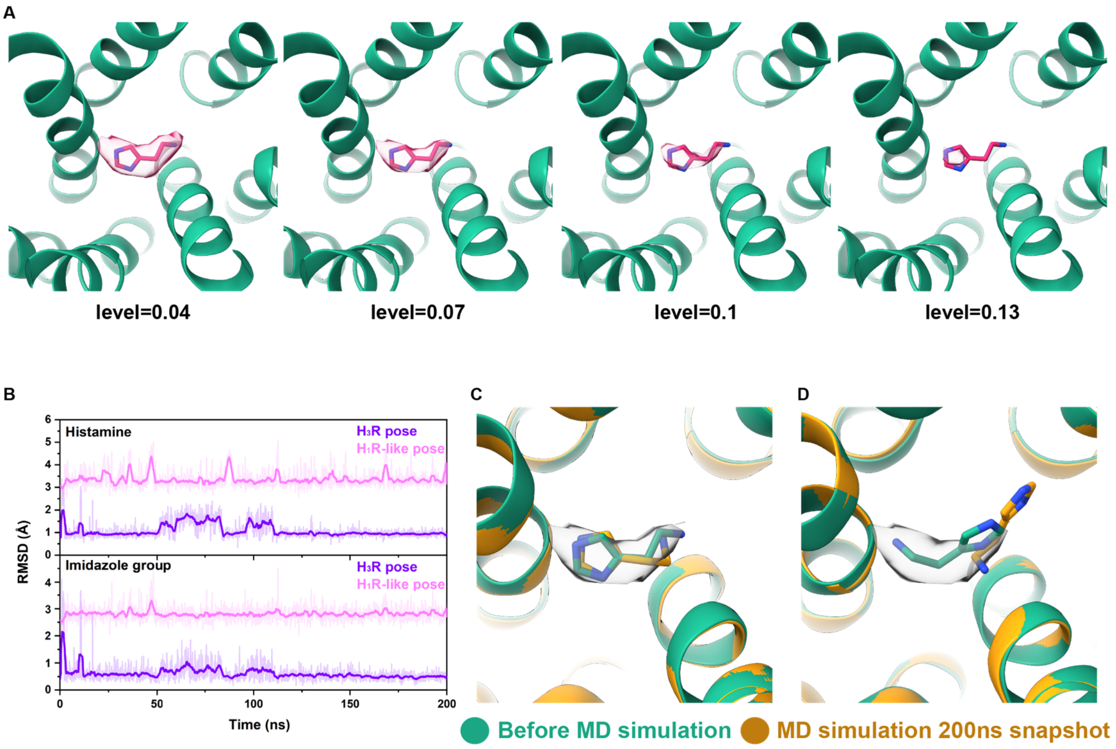

Figure S5. Cryo-EM density map and molecular dynamics simulations analyses of the specific pose of histamine bound to H_3R. A) Cryo-EM density map of histamine shown at the level of 0.04, 0.07, 0.1, and 0.13 of H_3R-histamine complex. B) RMSD analyses of H_3R-histamine 200ns trajectories based on the two poses of histamine. (Up) RMSDs of histamine in H_3R pose (purple) and H_1R-like pose (pink). (Down) RMSDs of the imidazole group of histamine. C-D) Superposition of H_3R-histamine models before MD simulations and 200 ns simulation snapshots of H_3R pose (C) and H_1R-like pose (D).

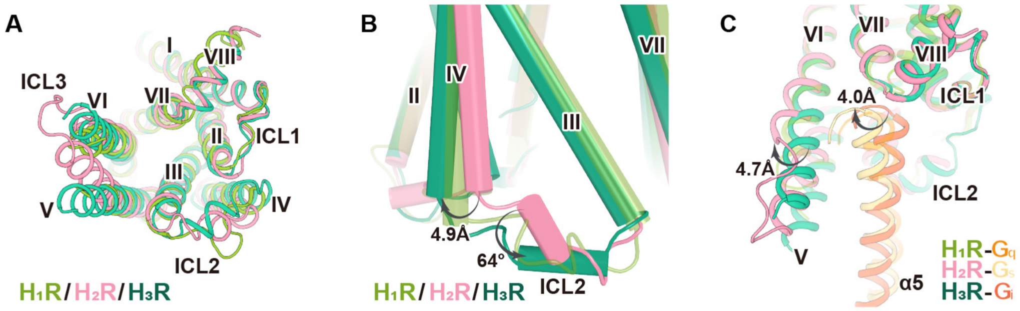

Figure S6. Structural comparison of histamine receptor-G complexes. A-B) Structural comparison of the cytoplasmic regions (A) and TM3-ICL2-TM4 regions (B) of H_1R, H_2R, and H_3R structures. C) Comparison of the interface between the α5-helix and receptor cytoplasmic cavity of H_1R-G_q, H_2R-G_s, and H_3R-G_i complexes.

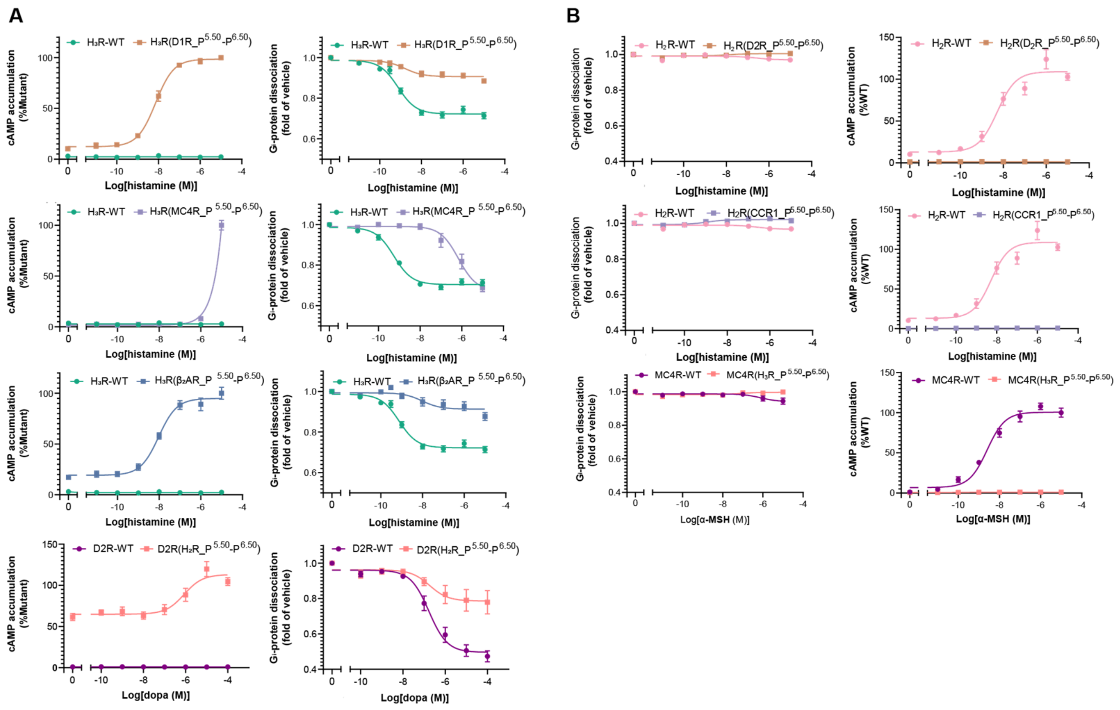

Figure S7. "TM5/TM6 swap" experiments in class A receptors. A-B) Dose-dependent curves for histamine induced cAMP accumulation and G-protein dissociation in HEK293 cells expressing chimera receptors (n =3). (A) The cAMP assay and NanoBit assay were performed on $H_3R(D1R\_P^{5.50}\text{-}P^{6.50})$, $H_3R(MC4R\_P^{5.50}\text{-}P^{6.50})$, $H3R(\beta_2AR\_P^{5.50}\text{-}P^{6.50})$, and $D2R(H_2R\_P^{5.5}\text{-}P^{6.5})$ to explore their G-protein selectivity. All chimera receptors gained the ability to activate the G_s signal pathway and lost the ability to dissociate G_i-protein. (B) The cAMP assay and NanoBit assay were performed on $H_2R(D2R\_P^{5.5}\text{-}P^{6.5})$, $H_2R(CCR1\_P^{5.5}\text{-}P^{6.5})$, and $MC4R(H_3R\_P^{5.5}\text{-}P^{6.5})$ to explore their G-protein selectivity. All chimera receptors lost the ability to activate the G_s signal pathway and did not gain the ability to dissociate G_i-protein.

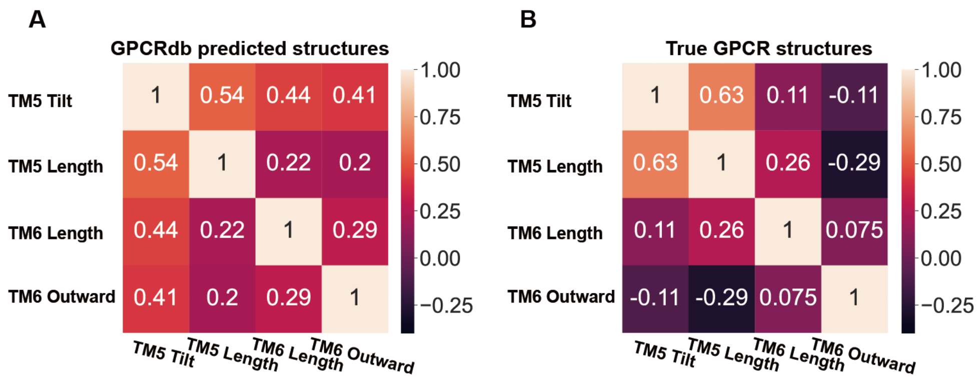

Figure S8. Correlation Matrix heatmaps. A-B) Heatmaps depicting the correlation matrix of GPCRdb structures (A) and True structures (B). Each element of the matrix represents the correlation between two features, with positive values indicating a positive correlation and vice versa. Notably, the TM6 Length in True structures exhibits a negative correlation with the TM5 tilt and TM5 Length, while in GPCRdb, they display a positive correlation.

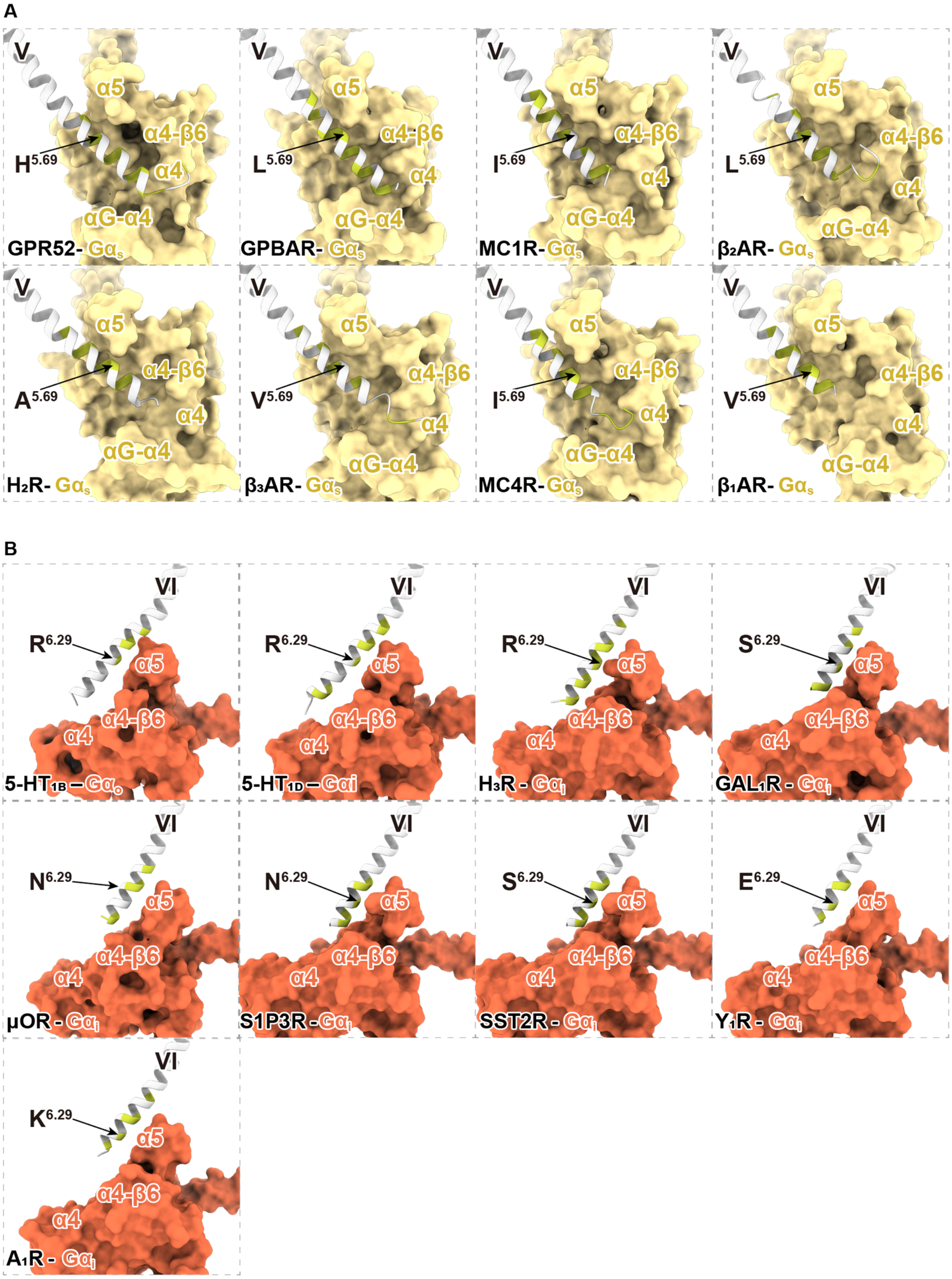

Figure S9. Interactions of Gα with TM5 or TM6. A) Binding surfaces of TM5 with $G\alpha_s$ of G_s-coupled receptors. Extended helices of TM5 beyond 5.69 are indicated. Residues that interact with Gα are heighted in green. B) Binding surfaces of TM6 with $G\alpha_i$ of G_i-coupled receptors. Extended helices of TM6 beyond 6.29 are indicated. Residues that interact with Gα are heighted in green.

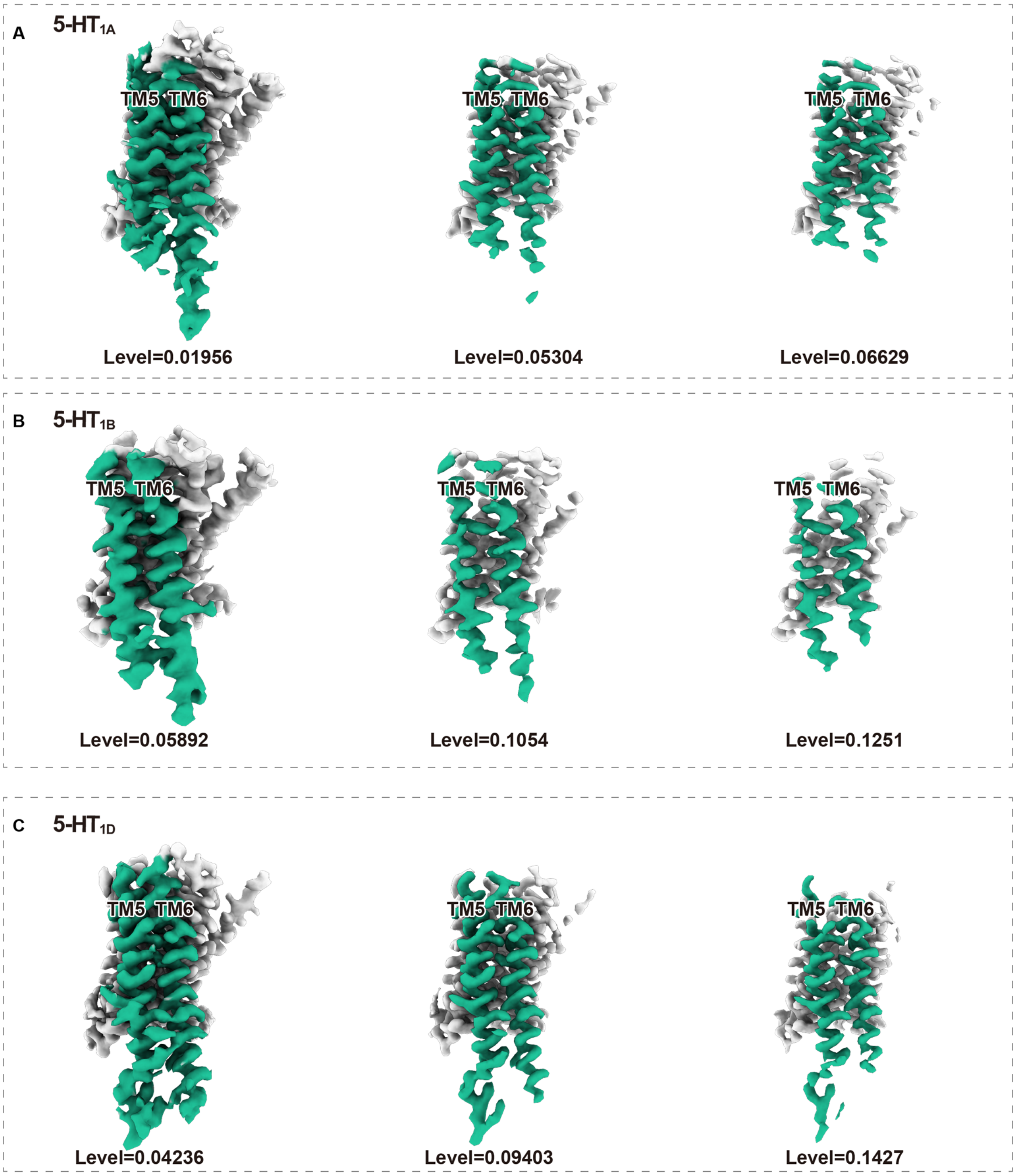

Figure S10. Cryo-EM density map of represent $G_{i/o}$-coupled complexes. A) Cryo-EM density map of TM5/TM6 of 5-HT_{1A} shown at the level of 0.01956, 0.05304, and 0.06629. B) Cryo-EM density map of TM5/TM6 of 5-HT_{1B} shown at the level of 0.05892, 0.1054, and 0.1251. C) Cryo-EM density map of TM5/TM6 of 5-HT_{1D} shown at the level of 0.04236, 0.09403, and 0.1427.

Table S1. Cryo-EM data collection, model refinement and validation statistics

	Amathmine–H_2R–G_s	Histamine–H_3R–G_s	Immepip–H_3R–G_s
**Data collection and processing**			
Magnification	4,9310	4,9310	4,9310
Voltage (kV)	300	300	300
Electron exposure (e^{-}/Å^2)	62	62	62
Defocus range (μm)	-0.5 ~ -2.5	-0.5 ~ -2.5	-0.5 ~ -2.5
Pixel size (Å)	1.014	1.014	1.014
Symmetry imposed	C1	C1	C1
Initial particle projections (no.)	3,361,930	2,487,329	2,050,983
Final particle projections (no.)	368,447	234,203	330,449
Map resolution (Å)	2.7	2.7	3.0
FSC threshold	0.143	0.143	0.143
Map resolution range (Å)	2.0-4.5	2.0-4.5	2.0-4.5
**Refinement**			
Initial model used	7CFM	7E32	7E32
Model resolution (Å)	2.9	2.7	3.0
FSC threshold	0.5	0.5	0.5
Map sharpening *B* factor (Å^2)	-107.3	-83.6	-106.8
Model composition			
Non-hydrogen atoms	8229	9037	9102
Protein residues	1041	1145	1145
Lipid	1	1	1
Water	0	0	0
B factors (Å^2)			
Protein	38.85	55.81	61.61
Lipids	59.48	101.56	86.71
R.m.s. deviations			
Bond lengths (Å)	0.005	0.005	0.015
Bond angles (°)	0.771	0.824	1.450
Validation			
MolProbity score	1.23	1.76	1.36
Clashscore	4.57	10.27	4.57
Rotamer outliers (%)	0.00	0.00	0.00
Ramachandran plot			
Favored (%)	98.15	96.55	97.34
Allowed (%)	1.85	3.45	2.66
Disallowed (%)	0	0.00	0.00

Table S2. Effects of mutations on histamine receptors at ligand binding pocket (H_2R-amthamine, H_3R-histamine, and H_3R-immepip respectively)

amthamine	pEC50±SEM[a,b]	Emax±SEM[a,b]	n	Expression (% WT)
H_2R-WT	8.78±0.26	99±1	3	100
H_2R-D98$^{3.32}$A	ND	ND	3	107.67±7.02
H_2R-C102$^{3.36}$A	6.79±0.17****	85.68±3.89	3	109.16±1.33
H_2R-T103$^{3.37}$A	6.19±0.66****	30.58±4.88***	3	108.28±14.60
H_2R-D186$^{5.42}$A	6.77±0.70****	38.55±4.58***	3	106.01±4.38
H_2R-T190$^{5.46}$A	8.36±0.44	65.30±3.72*	3	103.19±6.21
H_2R-Y250$^{6.51}$A	7.13±0.16****	84.89±4.44	3	101.25±12.81
H_2R-F251$^{6.52}$A	6.73±0.27****	71.61±11.60*	3	110.04±2.67
H_2R-F254$^{6.55}$A	ND	ND	3	99.98±6.29
H_2R-L274$^{7.39}$A	7.25±0.23****	108.91±8.24	3	102.20±5.53
**amthamine**	**pEC50±SEM[a,b]**	**Emax±SEM[a,b]**	**n**	**Expression (% WT)**
H_2R-WT	7.91±0.06	99±1	3	100
H_2R-V99$^{3.33}$Y	ND	ND	3	100.33±4.04
H_2R-D186$^{5.42}$T	ND	ND	3	183.75±19.71
H_2R-T190$^{5.46}$N	ND	ND	3	15.99±4.85
**histamine**	**pEC50±SEM[a,b]**	**Emax±SEM[a,b]**	**n**	**Expression (% WT)**
H_3R-WT	9.10±0.05	-0.37±0.02	19	100
H_3R-D114$^{3.32}$A	NR	NR	3	111.88±9.69
H_3R-Y115$^{3.33}$A	ND	ND	3	85.69±8.45
H_3R-C118$^{3.36}$A	ND	ND	3	108.43±7.25
H_3R-T119$^{3.37}$A	8.87±0.11	-0.31±0.02	3	96.43±7.35
H_3R-E206$^{5.46}$A	NR	NR	3	97.49±6.65
H_3R-Y374$^{6.51}$A	ND	ND	4	91.57±5.13
H_3R-T375$^{6.52}$A	ND	ND	3	118.84±6.19
H_3R-F398$^{7.39}$A	ND	ND	5	98.59±2.45
H_3R-L401$^{7.42}$A	ND	ND	6	106.27±9.44
H_3R-W402$^{7.43}$A	NR	NR	4	88.47±6.65
**immepip**	**pEC50±SEM[a,b]**	**Emax±SEM[a,b]**	**n**	**Expression (% WT)**
H_3R-WT	10.15±0.08	-0.32±0.02	13	100
H_3R-D114$^{3.32}$A	NR	NR	3	111.88±9.69
H_3R-Y115$^{3.33}$A	10.02±0.09	-0.33±0.04	3	85.69±8.45
H_3R-C118$^{3.36}$A	8.61±0.16****	-0.31±0.04	3	108.43±7.25
H_3R-T119$^{3.37}$A	10.54±0.09*	-0.25±0.03	4	96.43±7.35
H_3R-E206$^{5.46}$A	NR	NR	3	97.49±6.65
H_3R-Y374$^{6.51}$A	8.54±0.13****	-0.32±0.03	3	91.57±5.13
H_3R-T375$^{6.52}$A	8.41±0.04****	-0.46±0.08*	3	118.84±6.19
H_3R-F398$^{7.39}$A	ND	ND	3	98.59±2.45
H_3R-L401$^{7.42}$A	7.96±0.03****	-0.30±0.04	3	106.27±9.44
H_3R-W402$^{7.43}$A	ND	ND	3	88.47±6.65

[a] Data shown are means ± SEM from at least three independent experiments performed in technical triplicate. *P<0.01; **P<0.001 and ***P<0.0001 by one-way ANOVA followed by Dunnett's multiple comparisons test, compared with the response of the WT.

[b] The Emax is defined as the window between the maximal response and the vehicle. ND, not determinable, which refers to cannot be established over the tested concentration range, such that an Emax was not reached and therefore span could not be calculated. NR refers to no response (or response < 10% WT) occurred as the concentration of ligand changes.

Table S3. Structure-based sequence alignment of aminergic receptors at position 3.32 and 5.46

Receptors		Residues		Receptors		Residues	
Uniport	IUPHAR	3.32	5.46	Uniport	IUPHAR	3.32	5.46
5HT1A	5-HT_{1A}	D	A	ADA1B	α_{1B}	D	S
5HT1B	5-HT_{1B}	D	A	ADA1D	α_{1D}	D	S
5HT1D	5-HT_{1D}	D	A	ADA2A	α_{2A}	D	S
5HT1E	5-HT_{1E}	D	A	ADA2B	α_{2B}	D	S
5HT1F	5-HT_{1F}	D	A	ADA2C	α_{2C}	D	S
5HT2A	5-HT_{2A}	D	S	ADRB1	β_1	D	S
5HT2B	5-HT_{2B}	D	A	ADRB2	β_2	D	S
5HT2C	5-HT_{2C}	D	A	ADRB3	β_3	D	S
5HT4R	5-HT_4	D	A	DRD1	D_1	D	S
5HT5A	5-HT_{5A}	D	A	DRD2	D_2	D	S
5HT6R	5-HT_6	D	T	DRD3	D_3	D	S
5HT7R	5-HT_7	D	A	DRD4	D_4	D	S
ACM1	M_1	D	A	DRD5	D_5	D	S
ACM2	M_2	D	A	HRH1	H_1	D	N
ACM3	M_3	D	A	HRH2	H_2	D	T
ACM4	M_4	D	A	HRH3	H_3	D	E
ACM5	M_5	D	A	HRH4	H_4	D	E
ADA1A	α_{1A}	D	S	TAAR1	TA_1	D	S

Table S4. Effects of mutations of histamine receptors at 5.46 position

	pEC50±SEM[a,b]	Emax±SEM[a,b]	n	Expression (% WT)
H_1R-WT	6.01±0.16	-0.254±0.03	4	100
H_1R-N198$^{5.46}$E	ND	ND	4	102.40±8.92
	**pEC50±SEM[a,b]**	**Emax±SEM[a,b]**	**n**	**Expression (% WT)**
H_3R-WT	9.13±0.08	-0.39±0.04	6	100
H_3R-E206$^{5.46}$A	NR	NR	3	97.49±6.65
H_3R-E206$^{5.46}$ N	ND	ND	3	105.72±11.49
H_3R-E206$^{5.46}$ D	NR	NR	4	79.63±15.21

[a] Data shown are means ± SEM from at least three independent experiments performed in technical triplicate. *P<0.01; **P<0.001 and ***P<0.0001 by one-way ANOVA followed by Dunnett's multiple comparisons test, compared with the response of the WT.

[b] The Emax is defined as the window between the maximal response and the vehicle. ND, not determinable, which refers to cannot be established over the tested concentration range, such that an Emax was not reached and therefore span could not be calculated. NR refers to no response (or response < 10% WT) occurred as the concentration of ligand changes.

Table S5. Characteristics of True GPCR datasets

Primary G protein signaling	Receptors	PDB ID	Gα subtype	Last TM5 residue	Last TM5 number	Last TM6 residue	Las TM5 number	TM5 length	TM6 length	TM5 tilt	TM6 outward	Prediction Result
G_s	DRD1	7JV5	$G\alpha_s$	239	5.83	264	6.27	33	23	25.97	32.92	TRUE
	GPBAR	7CFN	$G\alpha_s$	206	5.79	213	6.24	29	26	12.53	11.40	TRUE
	GPR52	6LI3	$G\alpha_s$	243	5.79	266	6.36	29	14	22.43	18.73	TRUE
	MSHR	7F4H	$G\alpha_s$	225	5.76	238	6.32	26	18	12.37	23.15	TRUE
	GPR3		$G\alpha_s$	230	5.75	242	6.30	25	20	21.63	25.40	TRUE
	HRH2		$G\alpha_s$	219	5.75	229	6.30	25	20	18.84	31.91	TRUE
	ADRB2	7DHI	$G\alpha_s$	236	5.75	266	6.28	25	22	13.70	27.77	TRUE
	GPR6		$G\alpha_s$	262	5.74	273	6.29	24	21	18.40	32.02	TRUE
	ADRB3	7DH5	$G\alpha_s$	240	5.74	290	6.33	24	17	19.51	21.30	TRUE
	MC4R	7F53	$G\alpha_s$	227	5.73	239	6.29	23	21	8.24	28.21	TRUE
	5HT6R	7XTB	$G\alpha_s$	223	5.73	264	6.31	23	19	9.10	26.36	TRUE
	GPR101		$G\alpha_s$	226	5.72	397	6.33	22	17	16.21	23.20	TRUE
	ADRB1	7JJO	$G\alpha_s$	241	5.72	284	6.29	22	21	10.68	28.87	TRUE
	5HT7R	7XTC	$G\alpha_s$	273	5.72	325	6.33	22	17	3.78	16.69	FALSE
	PE2R4	7D7M	$G\alpha_s$	216	5.71	265	6.28	21	22	2.14	17.07	FALSE
	PE2R2	7CX3	$G\alpha_s$	228	5.69	259	6.30	19	20	2.82	7.70	FALSE
	AA2AR	6GDG	$G\alpha_s$	208	5.69	225	6.27	19	23	8.35	38.39	TRUE
	V2R	7DW9	$G\alpha_s$	234	5.67	264	6.28	17	22	4.25	9.28	FALSE
	FSHR	8I2G	$G\alpha_s$	556	5.65	564	6.27	15	23	7.21	32.84	TRUE
	LSHR	7FIH	$G\alpha_s$	553	5.65	564	6.30	15	20	7.64	23.30	FALSE
$G_{i/o}$	5HT1A	7E2Y	$G\alpha_{i1}$	227	5.70	325	6.15	20	35	5.61	28.81	TRUE
	5HT1B	6G79m	$G\alpha_o$	241	5.71	298	6.19	21	31	10.02	25.66	TRUE
	5HT1D	7E32	$G\alpha_{i1}$	229	5.70	285	6.19	20	31	5.61	27.09	TRUE
	HRH3		$G\alpha_{i1}$	234	5.74	344	6.21	24	29	0.00	20.82	TRUE
	OPRM	6DDE	$G\alpha_{i1}$	261	5.65	270	6.23	15	27	5.99	19.86	TRUE
	GALR1	7WQ3	$G\alpha_{i1}$	230	5.68	235	6.23	18	27	2.73	13.16	TRUE
	S1PR3	7EW2	$G\alpha_{i1}$	228	5.71	232	6.24	21	26	4.64	12.85	TRUE

SSR2	7T10	$G\alpha_{i3}$	237	5.67	245	6.24	17	26	3.95	23.20	TRUE
AA1R	7LD3	$G\alpha_{i2}$	212	5.70	225	6.26	20	24	4.57	25.27	TRUE
NPY1R	7VGX	$G\alpha_{i1}$	245	5.72	254	6.26	22	24	10.50	20.08	TRUE
S1PR5	7EW1	$G\alpha_{i1}$	222	5.68	243	6.27	18	23	4.59	28.17	TRUE
ADA2B	6K41	$G\alpha_{o}$	202	5.68	363	6.27	18	23	6.14	25.49	TRUE
CCR5	7F1S	$G\alpha_{i1}$	220	5.64	227	6.27	14	23	6.73	16.41	TRUE
CCR2	7XA3	$G\alpha_{i1}$	231	5.67	235	6.27	17	23	10.40	18.26	TRUE
CNR1	6N4B	$G\alpha_{i1}$	310	5.74	336	6.28	24	22	4.20	13.30	TRUE
ACM2	6OIK	$G\alpha_{o}$	212	5.64	380	6.28	14	22	3.23	20.91	TRUE
DRD2	7JVR	$G\alpha_{i1}$	224	5.73	366	6.28	23	22	9.43	14.71	TRUE
DRD3	7CMV	$G\alpha_{i1}$	222	5.72	322	6.28	22	22	6.12	16.51	TRUE
5HT1F	7EXD	$G\alpha_{i1}$	212	5.69	286	6.28	19	22	5.72	27.13	TRUE
CNR2	6PT0	$G\alpha_{i1}$	227	5.76	238	6.28	26	22	7.92	12.98	TRUE
5HT1E	7E33	$G\alpha_{i1}$	211	5.67	284	6.28	17	22	10.44	16.21	TRUE
CCR1	7VL9	$G\alpha_{i1}$	227	5.66	232	6.28	16	22	3.77	21.75	TRUE
CCR3	7X9Y	$G\alpha_{i1}$	228	5.67	232	6.28	17	22	11.54	13.97	TRUE
HCAR2	8J6P	$G\alpha_{i1}$	217	5.67	224	6.28	17	22	3.09	14.75	TRUE
S1PR1	7TD4	$G\alpha_{i1}$	234	5.71	249	6.28	21	22	3.23	16.44	TRUE
LPAR1	7TD0	$G\alpha_{i1}$	236	5.69	252	6.29	19	21	4.77	17.64	TRUE
CCR6	6WWZ	$G\alpha_{o}$	241	5.65	249	6.30	15	20	2.55	8.92	TRUE
CXCR2	6LFO	$G\alpha_{i1}$	240	5.67	246	6.30	17	20	9.71	6.51	TRUE
MRGX2	7S8M	$G\alpha_{i1}$	212	5.68	218	6.30	18	20	3.71	19.61	TRUE
MTR1A	7DB6	$G\alpha_{i1}$	216	5.67	234	6.31	17	19	3.51	49.34	TRUE
FPR1	7T6T	$G\alpha_{i1}$	231	5.68	237	6.31	18	19	8.99	17.80	TRUE
FPR2	6OMM	$G\alpha_{i1}$	231	5.68	238	6.32	18	18	3.33	15.21	TRUE
Q3KSP2	7JHJ	$G\alpha_{i1}$	211	5.64	222	6.32	14	18	5.65	24.28	TRUE
GPR88	7EJX	$G\alpha_{i1}$	228	5.74	283	6.34	24	16	8.26	17.72	TRUE

Table S6. Effects of swap of H$_2$R/H$_3$R with other GPCRs

**G$_i$-protein dissociation**				
	**pEC50±SEM[a,b]**	**Emax±SEM[a,b]**	**n**	**Expression (% WT)**
H$_2$R-WT	NR	NR	4	100
H$_2$R(H$_3$R_F$^{5.56}$-F$^{6.44}$)	NR	NR	3	29.23±4.01
H$_2$R(D$_2$R_P$^{5.50}$-P$^{6.50}$)	NR	NR	3	35.58±8.07
H$_2$R(CCR1_P$^{5.50}$-P$^{6.50}$)	NR	NR	3	34.40±4.41
	**pEC50±SEM[a,b]**	**Emax±SEM[a,b]**	**n**	**Expression (% WT)**
MC4R-WT	NR	NR	3	100
MC4R(H$_3$R_P$^{5.50}$-P$^{6.50}$)	NR	NR	3	65.09±12.42
**G$_s$-signal pathway activation**				
	**pEC50±SEM[a,b]**	**Emax±SEM[a,b]**	**n**	**Expression (% WT)**
H$_2$R-WT	8.26±0.07	99±1	4	100
H$_2$R(H$_3$R_F$^{5.56}$-F$^{6.44}$)	NR	NR	3	29.23±4.01
H$_2$R(D$_1$R_P$^{5.50}$-P$^{6.50}$)	NR	NR	3	35.58±8.07
H$_2$R(CCR1_P$^{5.50}$-P$^{6.50}$)	NR	NR	3	34.40±4.41
	**pEC50±SEM[a,b]**	**Emax±SEM[a,b]**	**n**	**Expression (% WT)**
MC4R-WT	8.55±0.11	99±1	3	100
MC4R(H$_3$R_P$^{5.50}$-P$^{6.50}$)	NR	NR	3	65.09±12.42
**G$_s$-signal pathway activation**				
	**pEC50±SEM[a,b]**	**Emax±SEM[a,b]**	**n**	**Expression (% WT)**
H$_3$R-WT	NR	NR	3	100
H$_3$R(H$_2$R_P$^{5.50}$-P$^{6.50}$)	7.86±0.11	99±1	3	37.32±7.34
H$_3$R(D$_1$R_P$^{5.50}$-P$^{6.50}$)	8.10±0.19	99±1	3	54.17±6.30
H$_3$R(MC4R_P$^{5.50}$-P$^{6.50}$)	ND	ND	3	58.44±6.64
H$_3$R(β$_2$AR_P$^{5.50}$-P$^{6.50}$)	8.05±0.04	99±1	3	52.00±6.25
	**pEC50±SEM[a,b]**	**Emax±SEM[a,b]**	**n**	**Expression (% WT)**
D$_2$R-WT	NR	NR	3	100
D$_2$R(H$_2$R_P$^{5.50}$-P$^{6.50}$)	-6.10±0.02	99±1	3	23.65±0.12
**G$_i$-protein dissociation**				
	**pEC50±SEM[a,b]**	**Emax±SEM[a,b]**	**n**	**Expression (% WT)**
H$_3$R-WT	9.14±0.10	-0.40±0.04	7	100
H$_3$R(H$_2$R_P$^{5.50}$-P$^{6.50}$)	9.35±0.24	-0.18±0.02**	3	37.32±7.34
H$_3$R(D$_1$R_P$^{5.50}$-P$^{6.50}$)	8.69±0.15	-0.09±0.02***	4	54.17±6.30
H$_3$R(MC4R_P$^{5.50}$-P$^{6.50}$)	5.76±0.10****	-0.35±0.02	3	58.44±6.64
H$_3$R(β$_2$AR_P$^{5.50}$-P$^{6.50}$)	-8.60±0.29	-0.21±0.06**	3	52.00±6.25
	**pEC50±SEM[a,b]**	**Emax±SEM[a,b]**	**n**	**Expression (% WT)**
D$_2$R-WT	6.88±0.30	-0.50±0.06	4	100
D$_2$R(H$_2$R_P$^{5.50}$-P$^{6.50}$)	6.72±0.16	-0.30±0.08	3	23.65±0.12

[a] Data shown are means ± SEM from at least three independent experiments performed in technical triplicate. *P<0.01;

**P<0.001 and ***P<0.0001 by one-way ANOVA followed by Dunnett's multiple comparisons test, compared with the response of the WT.

[b] The Emax is defined as the window between the maximal response and the vehicle. ND, not determinable, which refers to cannot be established over the tested concentration range, such that an Emax was not reached and therefore span could not be calculated. NR refers to no response (or response < 10% WT) occurred as the concentration of ligand changes.

Table S7. Characteristics of activated GPCR structures from GPCRdb datasets

Primary G protein signaling	Uniport	Last TM5 residue	Last TM5 number	Last TM6 residue	Las TM5 number	TM5 length	TM6 length	TM5 tilt	TM6 outward
	5HT4R	236	5.82	249	6.25	32	25	22.89	34.74
	5HT6R	225	5.75	257	6.24	25	26	4.88	26.00
	5HT7R	274	5.73	316	6.24	23	26	6.05	25.46
	AA2AR	213	5.74	219	6.21	24	29	13.12	4.38
	AA2BR	218	5.74	223	6.24	24	26	7.71	3.81
	ACTHR	203	5.68	209	6.25	18	25	5.59	28.70
	ADRB1	260	5.74	313	6.24	24	26	4.06	30.70
	ADRB2	236	5.75	262	6.24	25	26	6.35	36.78
	ADRB3	242	5.76	282	6.25	26	25	23.68	25.31
	FSHR	556	5.65	564	6.27	15	23	5.73	26.58
	HRH2	216	5.72	222	6.30	22	20	6.85	26.97
G_s	LPAR6	212	5.66	219	6.25	16	25	8.03	16.65
	MC3R	221	5.72	234	6.27	22	23	9.23	30.64
	MC5R	220	5.73	232	6.29	23	21	3.73	4.67
	MSHR	221	5.72	235	6.29	22	21	5.57	28.38
	PD2R	241	5.79	255	6.25	29	25	1.76	8.78
	TAAR1	231	5.79	241	6.25	29	25	20.90	35.70
	TAAR2	243	5.72	250	6.23	22	27	13.03	26.36
	TAAR3	237	5.78	245	6.24	28	26	14.09	25.91
	TAAR5	233	5.72	238	6.21	22	29	9.77	19.54
	TAAR6	239	5.80	246	6.23	30	27	14.79	31.63
	TAAR8	237	5.79	246	6.24	29	26	11.21	23.88
	TAAR9	238	5.79	246	6.23	29	27	17.29	51.64
	TSHR	609	5.66	616	6.27	16	23	5.90	26.43
	5HT5A	235	5.73	274	6.24	23	26	3.62	24.90
	AA3R	211	5.72	218	6.23	22	27	6.17	29.07
	ADA2A	247	5.74	377	6.23	24	27	9.70	30.53
	ADA2C	247	5.75	371	6.24	25	26	18.09	28.24
	AGTR2	240	5.67	246	6.25	17	25	4.80	24.54
	APJ	235	5.72	243	6.30	22	20	11.09	20.51
	C3AR	365	5.67	373	6.31	17	19	3.53	20.71
	C5AR1	231	5.67	238	6.31	17	19	3.25	20.03
	CCR10	234	5.65	241	6.29	15	21	3.81	6.57
	CCR1	228	5.67	232	6.28	17	22	9.53	18.30
	CCR2	231	5.67	237	6.29	17	21	9.93	17.96
	CCR3	228	5.67	232	6.28	17	22	9.77	18.91
$G_{i/o}$	CCR4	231	5.67	235	6.28	17	22	10.28	16.46
	CCR7	250	5.65	258	6.30	15	20	1.19	6.20
	CCR8	227	5.67	231	6.28	17	22	10.73	17.77
	CCR9	241	5.65	249	6.30	15	20	2.68	9.08
	CLTR1	218	5.67	224	6.26	17	24	8.25	19.58
	CML1	249	5.67	256	6.31	17	19	3.06	20.25
	CX3C1	220	5.67	224	6.28	17	22	9.53	16.70
	CXCR1	231	5.67	237	6.30	17	20	9.54	6.26
	CXCR3	244	5.67	250	6.30	17	20	12.18	8.89

CXCR4	226	5.65	235	6.31	15	19	2.85	7.17
CXCR5	247	5.67	254	6.30	17	20	11.20	8.86
CXCR6	218	5.65	226	6.30	15	20	1.87	6.83
DRD4	229	5.75	385	6.26	25	24	6.29	20.22
EDNRA	288	5.71	296	6.29	21	21	1.63	18.78
EDNRB	307	5.72	313	6.25	22	25	4.24	19.53
FFAR2	208	5.67	214	6.27	17	23	7.39	18.92
FFAR3	213	5.67	219	6.29	17	21	3.47	14.32
FFAR4	244	5.75	269	6.24	25	26	6.63	18.86
FPR1	230	5.67	237	6.31	17	19	3.49	20.50
FPR2	231	5.68	238	6.32	18	18	10.42	20.82
FPR3	230	5.67	237	6.31	17	19	2.93	20.16
GALR1	234	5.72	242	6.30	22	20	9.86	17.03
GALR3	215	5.69	224	6.24	19	26	7.53	12.90
HCAR1	205	5.67	212	6.27	17	23	3.65	14.14
HCAR2	216	5.66	222	6.26	16	24	4.30	14.54
HCAR3	216	5.66	223	6.27	16	23	3.60	14.27
HRH4	209	5.73	293	6.25	23	25	4.74	24.27
LPAR1	239	5.72	247	6.24	22	26	9.91	23.10
LPAR2	224	5.74	229	6.23	24	27	7.38	23.97
LPAR3	220	5.72	228	6.24	22	26	9.48	22.44
LT4R1	211	5.68	217	6.31	18	19	8.24	18.96
LT4R2	241	5.67	245	6.25	17	25	9.19	10.28
MTR1B	234	5.72	240	6.24	22	26	9.14	57.43
NPBW1	231	5.66	240	6.24	16	26	6.18	16.66
NPBW2	242	5.68	249	6.24	18	26	7.85	12.55
NPFF1	245	5.67	258	6.23	17	27	14.86	20.75
NPFF2	356	5.73	365	6.24	23	26	15.42	18.77
NPY1R	245	5.72	250	6.22	22	28	13.39	17.68
NPY2R	250	5.69	257	6.24	19	26	12.36	17.78
NPY4R	246	5.70	255	6.25	20	25	14.05	14.48
OPRD	245	5.70	250	6.24	20	26	6.72	18.06
OPRK	254	5.66	262	6.23	16	27	4.46	15.24
OPRX	244	5.67	251	6.23	17	27	4.24	14.33
OXER1	280	5.66	288	6.28	16	22	3.77	11.42
P2Y12	223	5.72	230	6.29	22	21	16.36	9.17
P2Y13	241	5.71	252	6.32	21	18	19.75	6.64
P2Y14	219	5.71	228	6.30	21	20	11.67	13.10
PD2R2	236	5.68	242	6.31	18	19	2.26	21.75
PE2R3	264	5.73	271	6.24	23	26	4.60	12.55
PTAFR	215	5.67	220	6.23	17	27	9.96	15.93
QRFPR	255	5.79	263	6.25	29	25	29.11	15.63
RL3R1	308	5.79	315	6.24	29	26	16.51	16.37
RL3R2	233	5.67	239	6.28	17	22	7.55	18.65
S1PR3	228	5.71	232	6.24	21	26	3.80	9.12
S1PR4	233	5.68	239	6.23	18	27	8.85	16.72
SSR1	252	5.67	259	6.23	17	27	5.95	14.60
SSR2	236	5.66	244	6.23	16	27	0.31	13.31
SSR3	239	5.71	247	6.25	21	25	6.81	14.64

SSR4	240	5.67	247	6.23	17	27	5.02	13.76
SSR5	234	5.71	244	6.31	21	19	7.30	2.12
SUCR1	216	5.67	221	6.24	17	26	8.38	9.00
XCR1	214	5.67	219	6.29	17	21	10.55	7.21

Table S8. Comparison of classifiers trained on the standardized geometries (TM5 length, TM5 tilt, TM6 outward) with and without labels shuffled

	no PCA[b]					
	precision[c]	recall	f1	accuracy	AUC	MCC
Training Phase[a]	77.43%	75.82%	0.7463	82.31%	0.8910	0.5285
Testing Phase	93.59%	87.50%	0.8943	90.74%	0.8162	0.8086
	no PCA, labels shuffled					
	precision	recall	f1	accuracy	AUC	MCC
Training Phase	42.12%	49.29%	0.4457	71.95%	0.4489	-0.0140
Testing Phase	47.25%	49.74%	0.4192	61.33%	0.3485	-0.0127

[a]For the training phase, the average metrics in 25 repeats of 10-fold cross-validation are reported. For the testing phase, the best result is reported.

[b]The final model is trained on the three raw features (TM6 length excluded) of the GPCRdb dataset rather than in the PCA space.

[c]The precision, recall and f1-score are reported in macro average. The AUC is the Area-Under-Curve (AUC) of Receiver Operating Characteristic (ROC) curve. The MCC is the Matthews Correlation Coefficient.

Table S9. Enlarged interact area by extended TM5 or TM6

Primary G protein signaling	Receptors	PDB ID	Extended TM5 length (below 5.69)	Extended TM6 length (below 6.29)	Enlarged area ($Å^2$)
Gs	PE2R2	7CX3	19		7.46
	AA2AR	6GDG	19		48.59
	PE2R4	7D7M	21		29.46
	ADRB1	7JJO	22		168.51
	5HT7R	7XTC	22		165.12
	GPR101		22		150.38
	MC4R	7F53	23		180.59
	5HT6R	7XTB	23		108.44
	ADRB3	7DH5	24		151.39
	GPR6		24		306.59
	HRH2		25		243.00
	ADRB2	7DHI	25		222.38
	GPR3		25		311.54
	MSHR	7F4H	26		301.35
	GPBAR	7CFN	29		426.46
	GPR52	6LI3	29		433.47
	DRD1	7JV5	33		560.19
Gi/o	LPAR1	7TD0		21	63.50
	ACM2	6OIK		22	131.17
	CCR1	7VL9		22	84.65
	5HT1E	7E33		22	120.93
	CCR3	7X9Y		22	147.37
	HCAR2	8J6P		22	87.91
	5HT1F	7EXD		22	115.27
	S1PR1	7TD4		22	115.86
	DRD3	7CMV		22	163.38
	DRD2	7JVR		22	121.53
	CNR1	6N4B		22	82.16
	CNR2	6PT0		22	94.40
	CCR5	7F1S		23	91.91
	CCR2	7XA3		23	108.66
	S1PR5	7EW1		23	116.87
	ADA2B	6K41		23	106.97
	AA1R	7LD3		24	120.70
	NPY1R	7VGX		24	82.05
	SSR2	7T10		26	90.65
	S1PR3	7EW2		26	249.07
	OPRM	6DDE		27	131.96
	GALR1	7WQ3		27	293.18
	HRH3			29	311.53
	5HT1D	7E32		31	219.26
	5HT1B	6G79		31	174.84
	5HT1A	7E2Y		35	192.90

Table S10. Effects of truncation of histamine receptors at TM5/TM6

	**pEC50±SEM**[a,b]	**Emax±SEM**[a,b]	**n**	**Expression (% WT)**
H_2R-WT	8.38±0.10	99±1	6	100
H_2R-ΔI216$^{5.72}$-I219	7.80±0.12	102.50±15.56	4	56.27±10.72
H_2R-ΔQ212$^{5.68}$-I219	ND	ND	3	113.66±6.75
	**pEC50±SEM**[a,b]	**Emax±SEM**[a,b]	**n**	**Expression (% WT)**
H_3R-WT	9.05±0.03	-0.48±0.05	3	100
H_3R-ΔR274-S351$^{6.28}$	8.85±0.11	-0.49±0.05	3	95.59±12.81
H_3R-ΔR274-S352$^{6.29}$	9.13±0.08	-0.41±0.05	3	84.39±12.84

[a] Data shown are means ± SEM from at least three independent experiments performed in technical triplicate. *P<0.01; **P<0.001 and ***P<0.0001 by one-way ANOVA followed by Dunnett's multiple comparisons test, compared with the response of the WT.

[b] The Emax is defined as the window between the maximal response and the vehicle. ND, not determinable, which refers to cannot be established over the tested concentration range, such that an Emax was not reached and therefore span could not be calculated. NR refers to no response (or response < 10% WT) occurred as the concentration of ligand changes.

Table S11. Characteristics of activated class B1 GPCR structures from GPCRdb datasets

Receptor	Uniprot	family	pdb_code	primary	TM5 length	TM5 tilt	TM6 length	TM6 outward movement
CT receptor	CALCR	B1	8F2B	G_s	22	13.43	24	44.51
calcitonin receptor-like receptor	CALRL	B1	6UVA	G_s	20	11.76	21	42.08
CRF1 receptor	CRFR1	B1	6PB0	G_s	22	15.97	24	36.73
CRF2 receptor	CRFR2	B1	6PB1	G_s	22	15.27	24	37.01
GHRH receptor	GHRHR	B1	7V9M	G_s	18	14.26	20	38.57
GIP receptor	GIPR	B1	7FIN	G_s	22	18.83	23	44.29
GLP-1 receptor	GLP1R	B1	7DUQ	G_s	21	15.33	21	44.28
GLP-2 receptor	GLP2R	B1	7D68	G_s	21	18.37	24	42.84
glucagon receptor	GLR	B1	8FU6	G_s	20	15.1	20	41.93
PAC1 receptor	PACR	B1	8E3X	G_s	20	12.07	21	44.74
PTH1 receptor	PTH1R	B1	6NBI	G_s	24	20.87	23	36.22
PTH2 receptor	PTH2R	B1	7F16	G_s	21	15.63	24	37.2
secretin receptor	SCTR	B1	6WZG	G_s	20	12.83	24	41.22
VPAC1 receptor	VIPR1	B1	8E3Y	G_s	20	13.92	20	39.85
VPAC2 receptor	VIPR2	B1	7WBJ	G_s	20	15.69	19	40.45
